# Intelligent Hydrogel Sensors for Health Monitoring and Therapeutic Intervention: From Material Design to Integrated Diagnosis and Treatment

**DOI:** 10.3390/gels12100919

**Published:** 2026-10-14

**Authors:** Di Chu, Qi Hou, Mingxin Li, Yuan Dong

**Affiliations:** 1 College of Laboratory Medicine, Jilin Medical University, Jilin 132013, China; 2 College of Biomedical Engineering, Jilin Medical University, Jilin 132013, China

**Keywords:** intelligent hydrogel, biocompatibility, biosensor, closed-loop diagnosis and treatment, precision medicine

## Abstract

Intelligent hydrogel sensors are integrated sensing devices employing stimuli-responsive hydrogels as the core functional sensing layers. Featuring tissue-mimetic mechanical flexibility, superior biocompatibility, and dynamic stimulus responsiveness, they fundamentally resolve the mechanical and biological interfacial mismatch between traditional rigid electronics and soft living tissues, providing new avenues for in situ, real-time, and in vivo physiological monitoring and precise biomedical intervention. This review systematically elaborates the full-scale design logic, multifunctional integration strategies, and cutting-edge biomedical applications of engineered intelligent hydrogel sensors. The material selection principles covering natural, synthetic, and hybrid polymer systems, multi-mode crosslinking strategies for three-dimensional network construction, and functional modification approaches for conductivity, biological recognition, self-healing, adhesion, and antifouling performance are comprehensively summarized. The sensing mechanisms that convert physical deformation and biochemical variations into quantifiable electrical or optical signals are classified and analyzed, including resistive/capacitive, piezoelectric, electrochemical, and optical transduction modes. This review strictly focuses on hydrogel-dominant sensing platforms and discusses their versatile applications in wearable physiological monitoring, implantable in vivo sensing, and in vitro biochemical diagnosis. Particular emphasis is placed on the core role of intelligent hydrogels in constructing “perception–feedback–treatment” closed-loop theranostic systems, which enable autonomous on-demand drug release or physical stimulation in response to pathological signal changes, achieving integrated diagnosis and therapy. Despite substantial laboratory progress, the clinical translation of intelligent hydrogel sensors still faces critical challenges, including insufficient long-term in vivo stability, biological contamination, uncontrollable immune responses, large-scale manufacturing difficulties, and poor performance consistency. This review further outlines future development directions based on artificial intelligence-assisted material design, biomimetic structural engineering, advanced 3D/4D printing manufacturing, and interdisciplinary integration. It is anticipated that intelligent hydrogel sensors will evolve into highly integrated, personalized, and autonomous intelligent healthcare units, promoting the paradigm shift from passive universal treatment to proactive, personalized, and predictive precision medicine.

## 1. Introduction

Growing population aging, increasing chronic disease prevalence, and the shortage of organ donations have imposed enormous global healthcare burdens, accelerating the rapid development of precision medicine [1,2,3]. Precision medicine aims to realize accurate disease prevention, efficient tissue repair, and active functional regeneration, which strongly relies on advanced biomaterials and smart devices capable of dynamic bio-interface adaptation, long-term stable physiological monitoring, and precise intervention [4,5,6,7]. Traditional silicon-based and metal-based bioelectronic devices exhibit excellent signal processing capability but suffer from inherent mechanical rigidity, which is incompatible with the soft, humid, and dynamic characteristics of biological tissues [8,9]. Such interfacial mismatch frequently induces foreign body reactions, fibrous capsule encapsulation, severe signal attenuation, and adverse wearing discomfort, severely limiting their long-term wearable and implantable applications [10,11,12]. Therefore, developing new interface materials that can “seamlessly” integrate with biological tissues is the key to breaking through the bottleneck [13,14].

Hydrogels, featuring extracellular matrix (ECM)-mimicking three-dimensional hydrophilic networks, high water permeability, tissue-mimetic softness, and outstanding biocompatibility, have emerged as core interfacial and functional materials for next-generation biomedical devices [15,16,17]. However, it is critical to distinguish that not all hydrogel-integrated devices qualify as “intelligent hydrogel sensors”. In lateral flow test strips, surface plasmon resonance (SPR) substrates, and certain conventional wearable electrodes, hydrogels typically act merely as passive sample pads, immobilized coatings, or flexible carriers without participating in the core signal transduction process [18,19]. The “intelligent hydrogel sensors” defined in this review possess a core characteristic: the hydrogel matrix itself acts as the functional core, exhibiting life-like behaviors including active sensing (e.g., strain, pH, and specific biomolecules), signal transduction (converting stimuli into readable electrical or optical signals), and even therapeutic execution (e.g., drug release). This represents the evolution of hydrogels from a “static compatibility” paradigm toward a “dynamic interaction” paradigm [20,21].

Notably, unlike conventional single-function biosensors that merely enable passive signal acquisition, intelligent hydrogel sensors integrate passive health monitoring and active therapeutic intervention within a single system via rational structural and functional design. Specifically, the passive monitoring function relies on the intrinsic stimulus-responsive properties and integrated functional units of hydrogels, which can continuously capture subtle physical and biochemical signals from tissues and convert them into quantifiable electrical or optical data for real-time health evaluation. Once the monitored signals surpass the physiological threshold and indicate pathological abnormalities, the responsive hydrogel network triggers programmable structural changes, thereby activating downstream therapeutic modules including on-demand drug release, electrical stimulation, and mechanical regulation [22,23,24]. This distinctive material-derived switching mechanism enables a seamless transition from passive signal sensing to active targeted therapy, realizing the integrated closed-loop function of “sensing–feedback–therapy”.

This article focuses on the aforementioned strictly defined intelligent hydrogel sensors and their advanced applications in health monitoring and therapeutic intervention. It first systematically analyzes the design rationale for fundamental hydrogel building blocks, clarifying the distinctions between stimulus-responsive hydrogel systems and fully integrated sensor architectures. This review further elaborates on the rational strategies to upgrade primitive stimulus responses to quantitatively readable signal transduction capabilities via the integration of conductive components and biorecognition elements. Advanced functional modification approaches to self-healing, tissue adhesion, and anti-fouling properties are also comprehensively discussed. Subsequently, the underlying sensing mechanisms of intelligent hydrogel sensors are systematically illustrated, and their innovative applications in wearable, implantable, and in vitro diagnostic scenarios are summarized. Their functional roles across diverse application scenarios are further distinguished, specifically clarifying whether hydrogels act solely as a signal transduction interface or as an integrated perception and execution platform. Moreover, the pivotal role and feasible implementation strategies of intelligent hydrogels as core functional components for constructing closed-loop “perception–feedback–treatment” integrated diagnostic and therapeutic systems are highlighted. Finally, this review discusses the core translational challenges hindering the clinical transformation of hydrogel-based intelligent sensors from laboratory research to practical applications, and prospects the future development directions enabled by the deep integration of materials science, micro-nanotechnology, and artificial intelligence.

**Figure 1:**
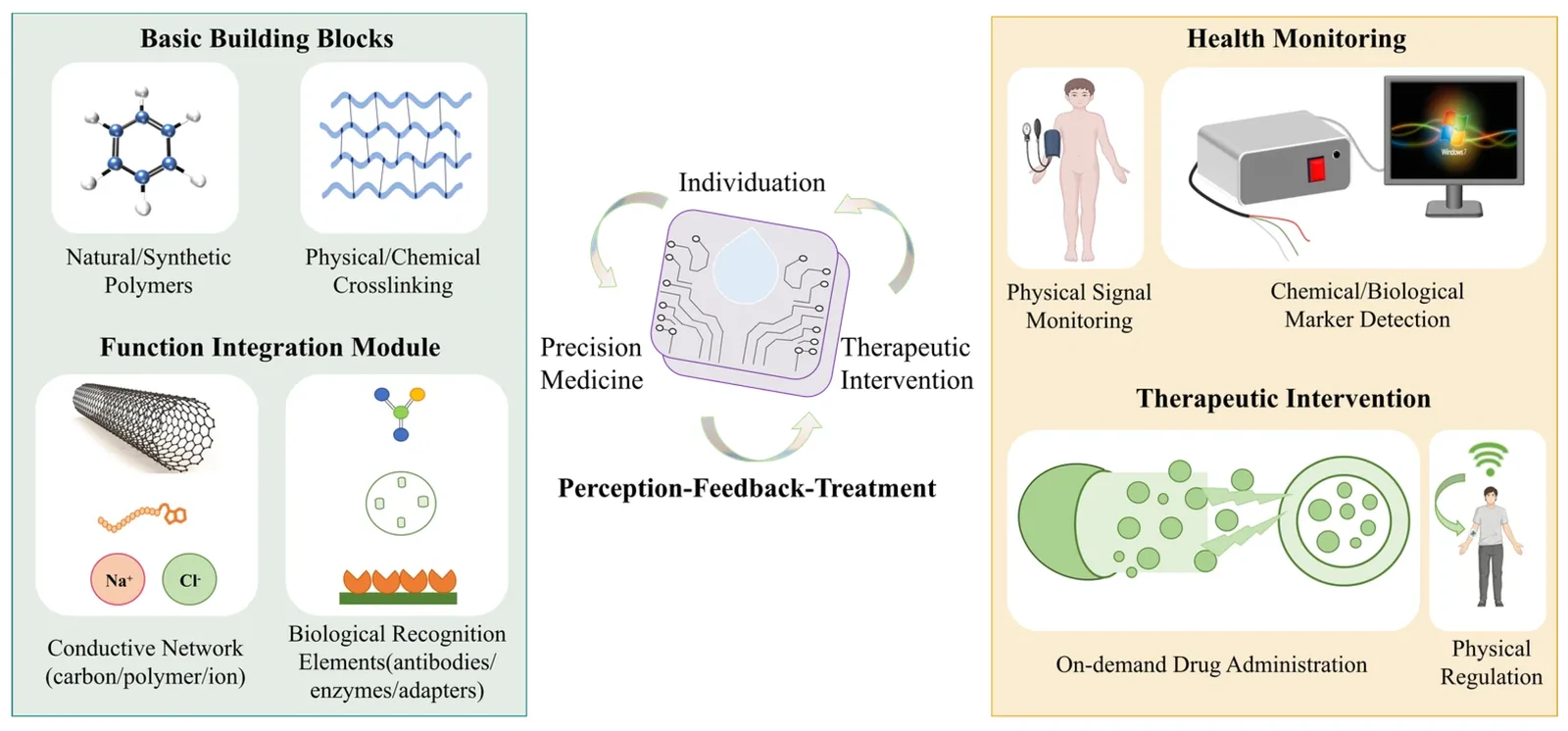
Intelligent hydrogel sensors: a comprehensive framework diagram from materials to integrated diagnosis and treatment.

To clarify the research scope of this review and strictly differentiate the functional roles of hydrogels in diverse devices, this article provides a systematic definition from four dimensions, including conceptual connotation, core characteristics, specific hydrogel functions, and the correspondence of these functions with the research focus of this review, as shown in Table 1. A comprehensive framework diagram is shown in Figure 1.

## 2. Design Foundation and Multifunctional Integration Strategy of Intelligent Hydrogel Sensors

The performance of intelligent hydrogel sensors is collectively determined by their composition, structure, and functionality. Starting from the rational selection of fundamental biomaterials, the systematic integration of conductive components, biological recognition elements, and specialized functional modules enables the transition of hydrogels from a “passive substrate” to an “active functional interface” [22,25,26,27,28].

### 2.1. Selection of Basic Biomaterials—The Foundation of Performance

**Figure 2:**
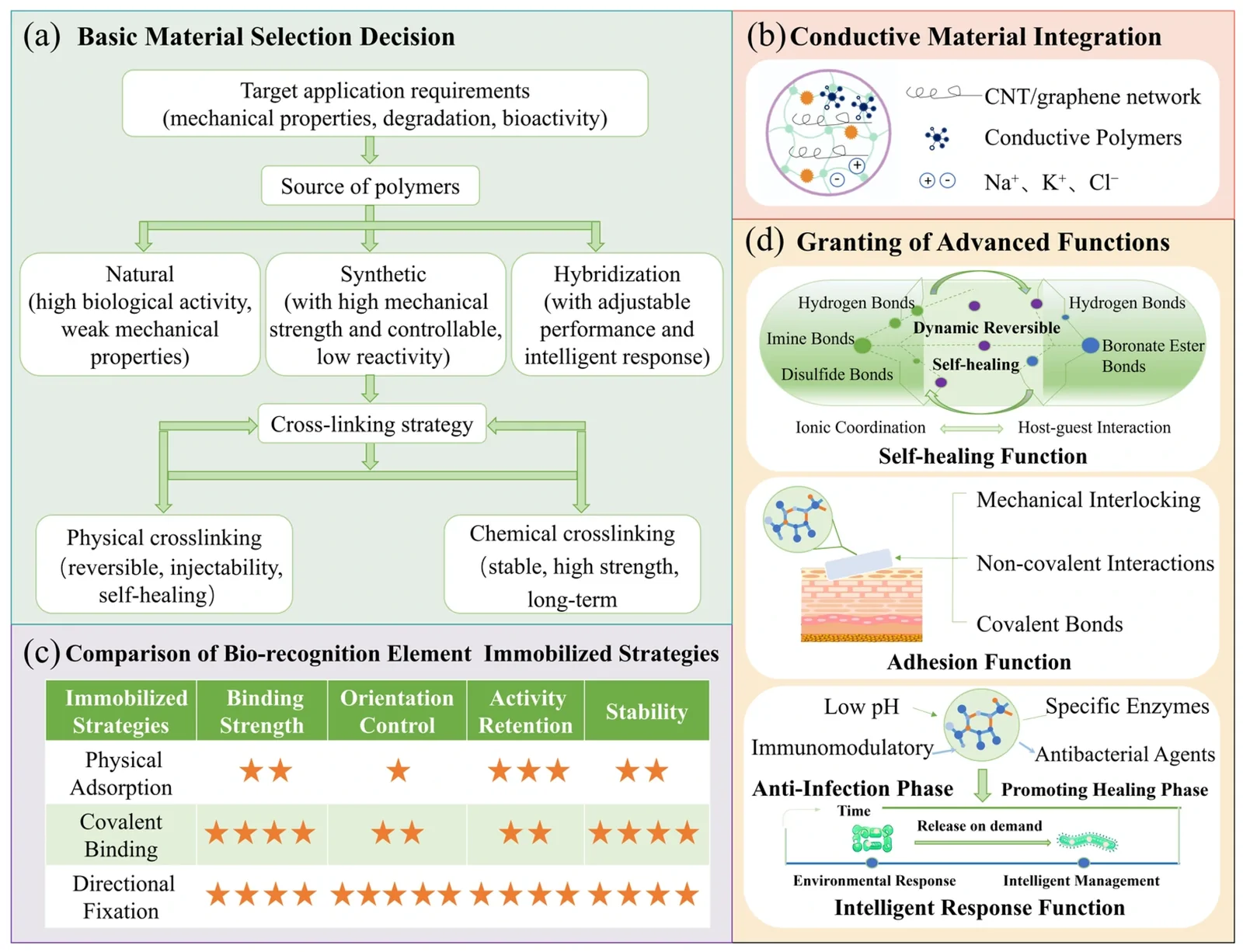
Design strategy of intelligent hydrogel sensors. (**a**) Basic material selection decision; (**b**) Conductive material integration; (**c**) Comparison of bio-recognition element immobilized strategiess; (**d**) Granting of advanced function.

The comprehensive performance of intelligent hydrogel sensors is fundamentally determined by their basic polymer components, crosslinking mechanisms, and functional group characteristics. Material selection for intelligent hydrogels follows a target-oriented rational design principle, which balances biological compatibility, mechanical adaptability, and functional responsiveness according to the requirements of specific biomedical scenarios [29,30,31]. The selection priority of natural, synthetic, and hybrid polymers depends on the matching degree between material intrinsic properties and application demands, rather than arbitrary material adoption [24,32,33]. The selection strategy of base materials is shown in Figure 2a.

Natural polymer hydrogels represented by hyaluronic acid, collagen, gelatin, sodium alginate, and chitosan possess inherent biological activity, excellent cytocompatibility, cell recognition capability, and degradability. Such unique biological advantages make them the preferred materials for tissue repair, wound monitoring, and short-term in vivo sensing applications. Nevertheless, natural hydrogels generally suffer from low mechanical modulus, poor structural stability, and uncontrollable degradation kinetics, which severely restrict their applications in long-term implantable sensing and high-load mechanical monitoring scenarios [34,35,36,37,38]. Synthetic polymer hydrogels, including polyvinyl alcohol (PVA), polyethylene glycol (PEG), and polyacrylamide (PAAm), feature highly controllable molecular structures, predictable mechanical properties, and facile functional modification. These merits endow synthetic hydrogels with outstanding performance repeatability and structural stability, making them suitable for standardized, long-term, and high-precision sensing platforms such as chronic drug delivery systems and stable wearable physiological monitoring. However, the lack of endogenous biological activity and potential inflammatory risks of partial degradation products limit their applications in highly active biological microenvironments and nerve-tissue interfacial sensing [39,40,41,42]. Hybrid hydrogels integrate the complementary advantages of natural and synthetic polymers through chemical grafting, copolymerization, or physical blending. By introducing synthetic rigid segments to compensate for the poor mechanical performance of natural matrices or embedding bioactive natural components to activate synthetic polymer networks, hybrid hydrogels achieve optimized trade-offs among biocompatibility, mechanical tunability, and intelligent responsiveness [43,44,45]. Benefiting from customizable stimulus-responsive structures, hybrid hydrogels can precisely convert subtle biological signals into macroscopic physical and chemical variations, exhibiting unique superiority in high-end intelligent sensing and closed-loop theranostic systems, such as neural interface monitoring and dynamic cartilage repair [46,47,48,49].

Crosslinking strategies construct the three-dimensional network of hydrogels and critically determine their structural stability, mechanical properties, and stimulus-responsive sensing performance [50,51]. Typical hydrogel crosslinking approaches are divided into physical crosslinking, conventional chemical crosslinking, and dynamic covalent crosslinking, which possess distinct structural features and application suitability for hydrogel biosensors.

Physical crosslinking relies on reversible non-covalent interactions including hydrogen bonds, ionic coordination, hydrophobic effects, and host–guest interactions to form hydrogel networks [52,53]. Its mild and reversible nature endows hydrogels with injectability, self-healing capacity, and stimulus responsiveness, suitable for minimally invasive sensing and repairable electronic skin devices. However, pure physical crosslinking provides limited mechanical strength and unstable network structure in complex physiological environments, restricting its utility in long-term and high-load biosensing [54,55,56].

Conventional chemical crosslinking is established via irreversible covalent bonding between polymer chains [57,58,59]. The permanent network delivers high mechanical strength and long-term structural integrity, enabling stable signal output under continuous mechanical load. Thus, chemically crosslinked hydrogels are widely used in long-term implantable sensors and durable tissue fillers requiring sustained stability. Nevertheless, irreversible covalent networks lack self-healing capability and flexibility, limiting their applications in dynamic and recyclable intelligent sensing scenarios [60,61,62].

Dynamic covalent crosslinking integrates the merits of physical and chemical crosslinking, emerging as a high-performance crosslinking strategy for advanced hydrogel sensors [63,64]. Typical dynamic bonds include Schiff base linkages, Diels–Alder adducts, and borate ester bonds. This strategy combines the high mechanical stability of chemical crosslinking and the reversible responsiveness of physical crosslinking, enabling hydrogels to simultaneously achieve robust mechanics, self-healing ability, and stimulus sensitivity. Such balanced performance greatly promotes the application of hydrogel biosensors in complex dynamic biological environments [65,66,67].

To systematically compare the differences among crosslinking strategies for biosensor development, their mechanisms, representative materials, technical advantages, and application limitations are summarized in Table 2.

### 2.2. Integration of Biocompatible Conductive Materials—Construction of Bioelectronic Interfaces

High-performance implantable and wearable bioelectronic sensors require dual core properties: stable electrical conductivity for physiological signal transduction and electrical stimulation, and superior biocompatibility for long-term dynamic coupling with biological tissues [68,69,70]. Different from traditional passive biocompatibility optimization that merely reduces foreign body reactions, the integration of multifunctional conductive materials realizes active functional upgrading of bio-interfaces, enabling hydrogel sensors to achieve synchronous signal sensing, stable electrical transmission, and adaptive tissue compatibility [71,72,73]. This section systematically elaborates the integration mechanisms, conductive principles, functional advantages, and typical application scenarios of carbon-based materials, conductive polymers, and ionic conductive systems, as well as the synergistic strategies for integrating biological recognition, self-healing, adhesion, and anti-fouling functionalities. The integration of multifunctional conductive materials is illustrated in Figure 2b.

Carbon-based conductive materials (graphene, carbon nanotubes) possess ultra-high intrinsic conductivity, large specific surface area, and excellent mechanical toughness [74,75]. Their conductive mechanism relies on the formation of continuous percolation networks through uniform filler dispersion and mutual contact within hydrogel matrices, which effectively improves the electrical transmission efficiency and structural rigidity of hydrogels [76,77]. Nevertheless, pristine carbon nanomaterials may cause inflammatory responses and poor biological durability. Targeted surface functionalization (PEGylation, biomolecule modification) can significantly improve tissue affinity and biological safety [78,79]. For representative examples, silk fibroin/graphene composite hydrogels integrate the cell-friendly microenvironment of silk fibroin and the high conductivity and mechanical reinforcement of graphene, which have been successfully applied to peripheral nerve repair conduits and cortical sensing electrode interfaces [80,81,82].

Conductive polymers including PEDOT: PSS and PANI exhibit unique ionic–electronic hybrid conduction characteristics and superior soft interface matching ability. Different from the single electronic conduction of carbon materials, conductive polymers can realize synchronous transmission of electrons and ions, which perfectly matches the ionic signal conduction mode of biological tissues [83,84,85]. They can be dispersed as nanofillers in hydrogel systems to build uniform conductive networks at low filling ratios and endow hydrogels with electrochemical sensing activity for biochemical molecule detection [86,87]. In addition, conductive polymer coatings can modify traditional rigid metal electrodes to reduce interface impedance, improve mechanical flexibility, and load bioactive factors, realizing high-sensitivity neural signal recording and tissue microenvironment regulation [88].

Ionic conductive hydrogels take hydrated polymer networks as the matrix and free mobile ions (Na^+^, K^+^, Cl^−^) as charge carriers, whose ionic conduction mechanism completely simulates the electrical signal transmission of skin and nerve tissues. Such biomimetic conduction mode endows ionic hydrogels with unparalleled bio-interface compatibility. By adjusting electrolyte concentration and polymer crosslinking density and introducing salting-out effects, the contradiction between high conductivity and mechanical softness can be effectively balanced. Benefiting from excellent stretchability, transparency, self-healing capability, and biocompatibility, ionically conductive hydrogels are widely used in flexible strain sensors, wearable physiological electrodes, and ionic–electronic coupling bio-interfaces [89,90,91].

### 2.3. Immobilization of Bio-Specific Recognition Elements

The high specificity of hydrogel biosensors originates from integrated biological recognition units, including enzymes, antibodies, antigens, and nucleic acid aptamers. Stable and high-activity immobilization of these biomolecules on hydrogel matrices or device surfaces is essential for constructing high-performance and long-term stable sensors. With the advancement of biomaterial modification and bionic technology, biomolecule immobilization strategies have evolved from simple physical adsorption to precise chemical coupling and biomimetic encapsulation, forming a systematic and multi-level technical system for biosensor fabrication [92,93]. Different immobilization strategies for bio-recognition elements are summarized in Figure 2c. The star ratings are evaluated based on four dimensions: binding strength, orientation control, activity retention, and stability, summarized from the published literature. More stars represent better performance in the corresponding indicator.

Traditional immobilization approaches mainly include physical adsorption, covalent binding, and encapsulation, all with inherent advantages and limitations [94]. Physical adsorption relies on weak non-covalent interactions (hydrogen bonds, van der Waals forces, hydrophobic effects) to immobilize biomolecules under mild conditions with negligible activity loss. However, weak interfacial adhesion easily causes biomolecule detachment in complex biological environments, resulting in poor sensor stability and reusability. Covalent binding creates robust chemical crosslinking between hydrogel functional groups and biomolecules, greatly improving immobilization stability, yet random crosslinking may damage biomolecular active centers and reduce sensing activity. Encapsulation traps biomolecules within hydrogel networks to avoid denaturation, but network confinement impedes substrate mass transfer and slows down sensing responses [95,96,97].

To address the drawbacks of traditional methods, advanced immobilization strategies including affinity immobilization, co-immobilization, and stimuli-responsive immobilization have been developed to achieve precise and controllable biomolecule fixation. Affinity immobilization utilizes specific biomolecular interactions for targeted immobilization, preserving the natural conformation and activity of recognition elements without random binding. Co-immobilization integrates functional enzymes with cofactors or synergistic enzymes to construct cascade catalytic systems, amplifying sensing signals and improving detection sensitivity. Differing from static traditional methods, intelligent responsive immobilization combines stimuli-responsive hydrogels with biomolecule fixation, dynamically tuning biomolecular activity via external pH and temperature variations to realize adaptive and high-precision sensing [98,99,100].

For antibody-based immunosensors, oriented immobilization is critical to maximize antigen-binding efficiency, which requires fully exposing antigen-binding Fab fragments to the detection solution. Traditional random covalent immobilization via amino–carboxyl crosslinking usually leads to disordered antibody arrangement and buried active sites, limiting sensor sensitivity. In contrast, directional immobilization strategies effectively solve this issue. The typical strategy employs Protein A/G to specifically bind antibody Fc segments, achieving ordered antibody immobilization with exposed Fab domains. Site-specific immobilization via antibody glycan oxidation also enables uniform antibody arrangement on aldehyde-modified hydrogel surfaces. These oriented immobilization approaches significantly improve the sensitivity and specificity of immunosensors [101,102].

Nucleic acid aptamers are single-stranded DNA/RNA molecules screened via SELEX technology, which exhibit high target-binding affinity and are regarded as “chemical antibodies” [103,104]. Benefiting from facile synthesis, good stability, easy modification, and low immunogenicity, aptamers are ideal recognition elements for high-performance hydrogel biosensors. Common aptamer immobilization methods include thiol–gold bonding, amino–carboxyl coupling, and biotin–avidin affinity immobilization. Advanced structural optimization strategies, such as cyclic structure design and multivalent assembly, can further enhance aptamer structural stability, resist nuclease degradation in biological fluids, and improve target-binding affinity and detection accuracy [105,106,107].

### 2.4. The Granting of Advanced Functions

The acquisition of advanced functionalities enables intelligent hydrogels to transcend their fundamental material properties via precise structural design, endowing them with organism-like capabilities including perception, decision-making, actuation, and adaptation [108,109], as illustrated in Figure 2d.

The self-healing capability of hydrogels relies on dynamically reversible non-covalent bonds (e.g., hydrogen bonds, ionic coordination interactions, and host–guest interactions) or dynamic covalent bonds (e.g., imine bonds, disulfide bonds, and borate ester bonds) [110,111]. Upon structural damage, hydrogels can accomplish autonomous repair via interfacial molecular diffusion and bond reformation. This functional characteristic substantially prolongs the service lifespan of wearable and implantable hydrogel devices. Cutting-edge studies have further incorporated chemical fuel-driven transient cross-linking strategies, endowing hydrogels with programmable and temporally tunable mechanical transformation behaviors [112,113].

Hydrogel adhesion is critical for establishing robust bonding with diverse wet biological tissues and device surfaces, which is indispensable for favorable biointegration [114]. Such adhesive behaviors originate from multiple interfacial interactions, including mechanical interlocking, non-covalent forces (hydrogen bonds, electrostatic interactions, hydrophobic effects, and π-π stacking), and covalent bonding (e.g., Schiff base reactions and Michael addition reactions) [115,116]. In particular, phenolic catechol chemistry represents a classic and well-established adhesion strategy. Beyond conventional approaches, the emerging strategy of soft stretchable topological adhesion employs sparsely crosslinked, ultra-long flexible polymer chains as adhesive layers. This design achieves a favorable combination of high adhesion strength and low interfacial stiffness, effectively addressing the longstanding trade-off wherein robust adhesion is usually accompanied by brittle interfaces and severe stress concentration [117,118,119].

Anti-biofouling capability, which inhibits the non-specific adsorption of proteins and cells in complex biological milieus, is essential for maintaining the long-term operational stability of hydrogel sensors [25]. Current antifouling strategies mainly fall into two categories: passive defense, which constructs hydrophilic polymer brushes and surface hydration layers to form a repulsive physical barrier, and active inhibition, which incorporates antibacterial components including silver nanoparticles, quaternary ammonium compounds, and photosensitizers to suppress microbial growth [49,120]. State-of-the-art hydrogel systems can intelligently respond to microenvironmental variations (e.g., low pH and specific enzymes at infection sites), release antibacterial agents and immunomodulatory factors on demand, and realize sequential wound management ranging from anti-infection treatment to tissue healing promotion [121,122].

## 3. Biomedical Sensing Mechanisms and Applications

### 3.1. Physical Signal Monitoring

**Figure 3:**
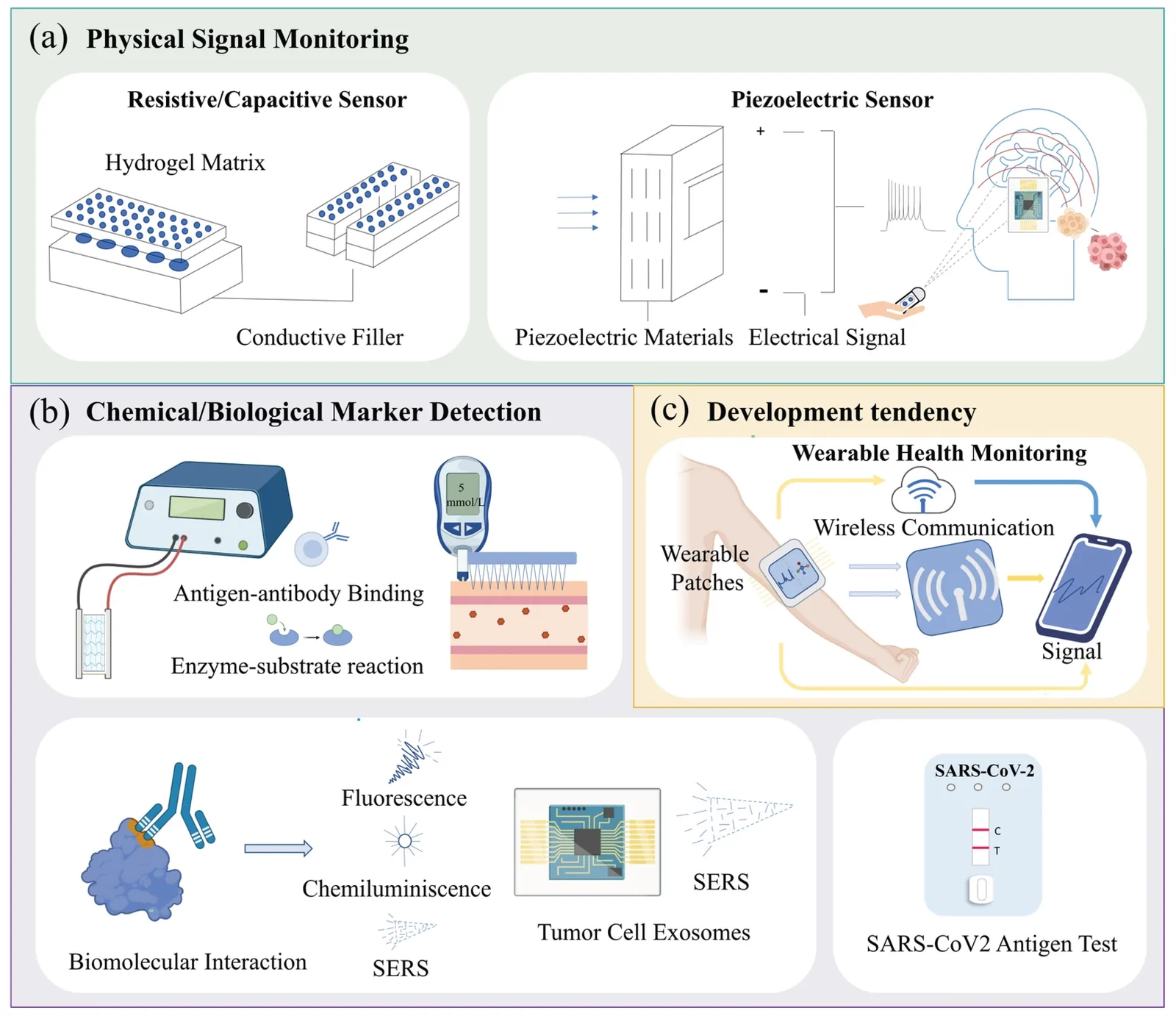
Biomedical sensing mechanisms and applications of intelligent hydrogel-based biomedical sensors.

Intelligent hydrogel sensors convert diverse bioactivity-derived signals into quantifiable and recordable electrical or optical signals via tunable alterations in their physical and chemical properties, as shown in Figure 3a.

Resistive and capacitive sensing [123,124,125,126] mechanisms are triggered by mechanical deformation of hydrogel sensors, particularly those integrated with conductive fillers. Stretching or compression reshapes internal conductive networks to alter electrical resistance, while variations in the thickness or area of the dielectric layer induce changes in capacitance. Based on this mechanism, flexible electronic skin can be integrated into clothing or directly attached to the skin, continuously monitoring pulse waves, respiratory rates, and joint movement postures [127,128]. For example, the composite material based on graphene/water gel is extremely sensitive to small strains and has been used to identify vocal cord muscle vibrations, enabling silent speech recognition [129].

Piezoelectric sensing [130,131] relies on piezoelectric materials such as polyvinylidene fluoride (PVDF) and zinc oxide (ZnO) nanowires to directly convert mechanical stress into electrical signals, eliminating the need for an external power supply [132,133]. Such superior characteristics render piezoelectric sensors highly applicable for implantable energy harvesting and self-powered sensing scenarios [134]. For example, piezoelectric sensors implanted near the heart can scavenge mechanical energy from cardiac contraction to sustain device operation while simultaneously monitoring intracardiac pressure [135].

Acoustic and mechanical sensing [136] perceives environmental physical variations by detecting changes in the propagation characteristics of sound waves within a medium [137,138]. A representative advance lies in the development of an implantable, tunable, ultrasound-driven MXene/PVA hydrogel device for tumor therapy. This device generates tailored alternating electromagnetic fields to disrupt the interstitial distribution of cancer cells while sparing adjacent normal neurons. Wireless ultrasonic actuation further substantially improves therapeutic efficiency. Such encouraging findings highlight the great application potential of this device for glioblastoma treatment and prognostic improvement [136].

### 3.2. Chemical/Biological Marker Detection

The detection of chemical and biological markers in body fluids provides critical biochemical information for evaluating human physiological and pathological states, enabling high-precision health monitoring and early disease warning. By analyzing specific biochemical molecules and biomarkers in diverse body fluids, including interstitial fluid, sweat, saliva, and blood, real-time and quantitative monitoring of bodily biochemical levels can be realized [139,140], as illustrated in Figure 3b. Based on the core sensing principles and signal transduction modes, the biomarker detection strategies of intelligent hydrogel sensors can be systematically classified into three mainstream categories: electrochemical sensing, optical sensing, and affinity sensing. Notably, the excellent tailorability of hydrogel network structures allows rational material modification and functional integration, which endows hydrogel sensing systems with specific molecular recognition capability and high detection accuracy, addressing the limitations of conventional sensing materials in biochemical detection.

Electrochemical sensing [141,142,143,144,145,146] is one of the most widely adopted biochemical detection techniques, which realizes biomarker quantification by capturing subtle variations in current, potential, or interfacial impedance induced by specific biological recognition and biochemical reactions (e.g., enzyme–substrate catalytic reactions, antigen–antibody specific binding) [147]. Different from traditional rigid electrochemical electrodes, functionalized intelligent hydrogels serve as ideal dual-functional substrates for electrochemical sensing, acting as both flexible conductive platforms and biocompatible immobilization matrices. The three-dimensional hydrophilic network of hydrogels can be modified via physical embedding, chemical crosslinking, and surface grafting strategies to stably load various biological recognition elements, including oxidases, antibodies, and specific enzymes. This structural modification not only maintains the biological activity of recognition elements by providing a hydrated and biocompatible microenvironment, but also effectively resists non-specific adsorption and biomolecular interference, achieving selective recognition of target biomarkers. For practical applications, continuous glucose monitoring represents the most mature commercial scenario of hydrogel-based electrochemical sensing. Microneedle hydrogel sensors implanted under the skin can specifically capture glucose molecules in tissue fluid and trigger enzymatic catalytic reactions, converting glucose concentration changes into measurable electrical signals for long-term, real-time glucose monitoring [148]. In neuroscience research, brain-implantable hydrogel microelectrode arrays with good tissue compliance adopt rapid scanning cyclic voltammetry technology. The modified hydrogel interface enables selective identification of neurotransmitters such as dopamine and serotonin, realizing real-time dynamic detection of neurotransmitter changes and providing effective technical support for exploring the pathological mechanisms of Parkinson’s disease, depression, and other neuropsychiatric disorders [149].

Optical sensing [150,151,152] relies on specific optical signal variations generated by the interaction between functionalized hydrogel interfaces and target biomolecules, including fluorescence emission, chemiluminescence response, and surface-enhanced Raman scattering (SERS) effects, to achieve high-sensitivity biochemical detection [153]. To endow hydrogels with selective optical recognition capability, hydrogel network structures can be functionally modified with fluorescent probes, SERS-active nanomaterials, and specific biorecognition ligands. Such modification enables the hydrogel sensing system to produce specific and amplified optical signal responses only when binding with target chemical/biological markers, while avoiding signal interference from interfering substances in complex body fluid environments. Lateral flow immunoassay based on functional hydrogel carriers is a typical portable optical sensing application. This technique integrates gene editing technology and hydrogel-based immunoassay strategies to realize rapid, sensitive, convenient, and highly specific biomarker detection. It has been successfully applied to severe acute respiratory syndrome coronavirus 2 antigen detection, greatly promoting the development of rapid in vitro diagnostic assays for COVID-19 [154]. In addition, emerging droplet microfluidic platforms integrate SERS-based chemical sensing units with modified hydrogel functional layers. The rationally designed hydrogel network achieves efficient enrichment and selective recognition of tumor cell exosomes and other key cancer markers, enabling rapid and ultra-sensitive detection of tumor biomarkers. This integrated system exhibits great application potential in early cancer screening and precise diagnosis [152,155].

Affinity sensing [156,157] is established on the basis of highly specific molecular interaction behaviors, including antigen–antibody specific binding and nucleic acid complementary base pairing [158]. As a typical representative of affinity sensing biosensors, surface plasmon resonance (SPR) biosensors modified with functional hydrogel layers possess superior molecular recognition performance. The hydrogel-modified sensing interface can stably immobilize specific affinity molecules and maintain their binding activity, realizing label-free, real-time dynamic monitoring of biomolecular interaction kinetics. Benefiting from the high selectivity and anti-interference ability of hydrogel functional layers, such affinity sensing platforms are widely applicable to new drug target screening, biomolecular mechanism research, and other basic biomedical research fields [159,160].

The representative hydrogel compositions, sensing mechanisms, target analytes, and key performance metrics of the above chemical/biological marker detection strategies are also compared in Table 3. The values in the table represent typical ranges, not precise values from a single reference. Overall, electrochemical sensors feature miniaturization capability and continuous monitoring feasibility but suffer from limited long-term stability. Optical sensors deliver high sensitivity and excellent multiplexing performance yet generally rely on bulky external readout setups. Affinity sensors enable label-free, real-time kinetic analysis but exhibit poor adaptability for in vivo continuous monitoring. These comparative analyses highlight the necessity of future research to comprehensively balance sensitivity, stability, and wearability, thereby facilitating the practical clinical translation of hydrogel sensing platforms.

### 3.3. Future Trends and Challenges

Future biomedical sensors are evolving from single-modal detection units to multi-modal integrated intelligent systems, as illustrated in Figure 3c. As a typical example, intelligent wearable patches can integrate electrocardiogram (ECG) electrodes for physical signal acquisition and sweat biochemical sensors for chemical signal detection, enabling comprehensive and multidimensional health status evaluation.

The ultimate development goal is to integrate hydrogel sensing platforms with microprocessors, wireless communication modules, and power supply units to construct closed-loop intelligent systems featuring “perception–analysis–transmission” and even advanced “perception–analysis–intervention” functional frameworks.

## 4. Beyond Perception: Towards Integrated Diagnosis and Treatment

**Figure 4:**
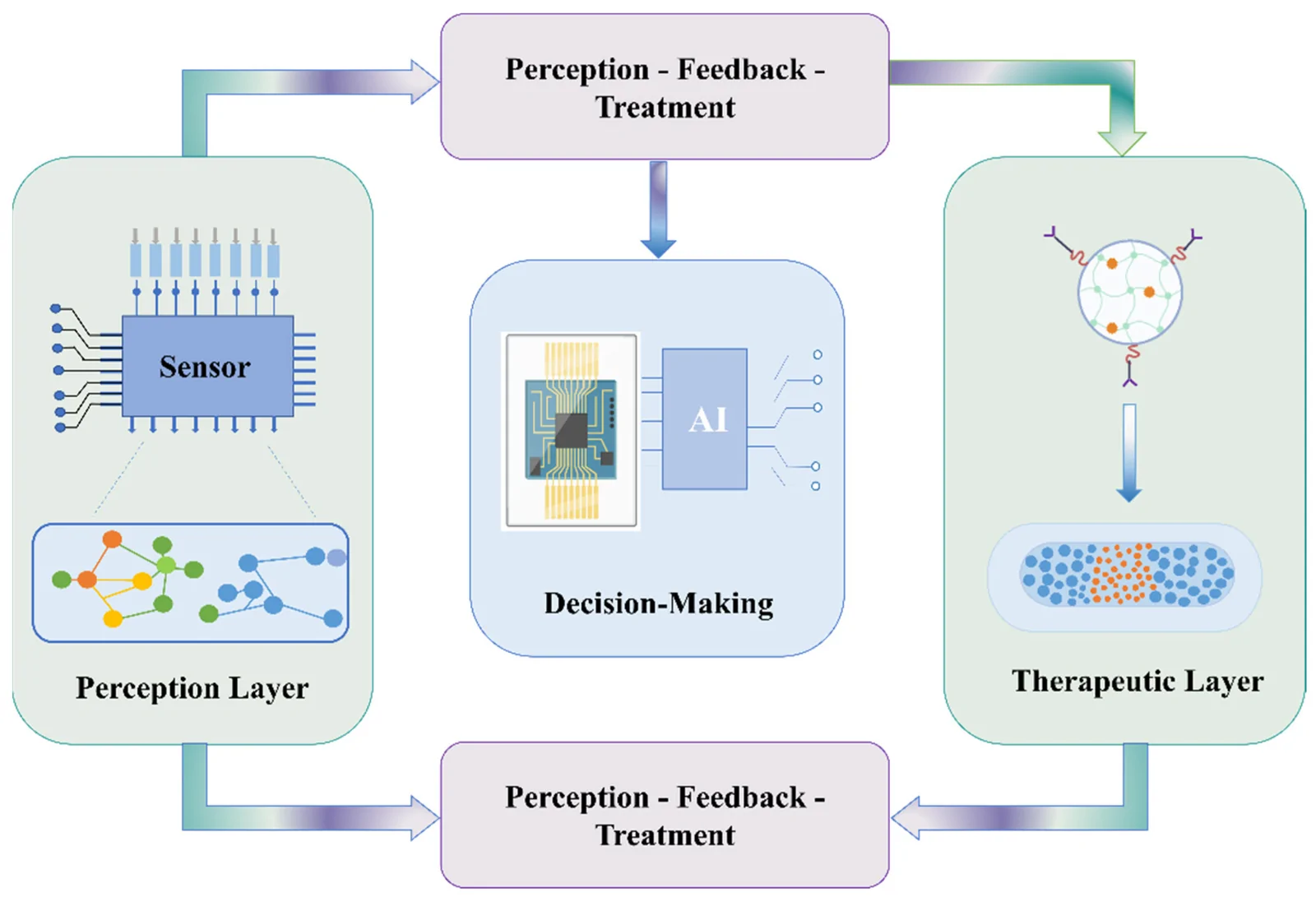
Diagram of intelligent hydrogel sensors for integrated diagnosis and treatment.

The ultimate value of intelligent hydrogel sensors lies in their dual capability for both diagnosis and treatment, enabling integrated theranostic functions. The core principle relies on the construction of a closed-loop “perception–feedback–treatment” system that mimics the intrinsic homeostatic regulatory mechanisms of the human body (such as blood sugar regulation), as shown in Figure 4.

High-performance sensing layers require sensors to integrate high sensitivity, superior specificity, excellent biocompatibility, and long-term stability. High sensitivity enables the capture of faint biological signals, as exemplified by graphene field-effect transistor-based sensors capable of single-protein-molecule detection [161]. High specificity is achieved via highly precise recognition elements, including engineered nucleic acid aptamers and molecularly imprinted polymers that serve as artificial antibodies [103]. Notably, synthetic biological circuits can further couple molecular recognition events with intracellular gene expression, realizing logical gating and inherent signal amplification capabilities. The feedback decision-making layer uses an internal or external microprocessor (or AI algorithm) to analyze and interpret the sensed data, determine the physiological/pathological state, and issue treatment instructions [162].

The therapeutic layer employs intelligent hydrogels as functional actuators to implement precise on-demand intervention. For example, once a local pH decrease within the tumor microenvironment or elevated concentrations of specific enzymes is detected, the hydrogel matrix undergoes swelling or degradation to rapidly release encapsulated anticancer drugs [163,164]. Alternatively, upon the detection of abnormal brain waves, the system can release neurotransmitters or deliver electrical stimulation to modulate neural activity [165].

This closed-loop system helps overcome the limitations of the previously prevalent, inefficient “diagnosis–treatment” separation model. In particular, it offers a promising disease management strategy for chronic diseases including diabetes, cardiovascular diseases, epilepsy, and other scenarios requiring rapid responses. Although the “perception–decision–treatment” closed-loop framework in terms of concepts provides an ideal blueprint for intelligent hydrogel sensors, its true value must be demonstrated through experimentally validated systems. Currently, several studies have successfully constructed complete hydrogel closed-loop systems and have verified the feasibility of “perception–feedback–treatment” at the in vitro or in vivo levels. This section will focus on analyzing these representative systems, conducting a systematic comparison from dimensions such as perception targets, perception mechanisms, decision/feedback components, treatment actuators, response times, and the degree of in vivo validation, as shown in Table 4.

## 5. Current Challenges and Future Prospects

### 5.1. The Core Challenges Faced by Translational Medicine

**Figure 5:**
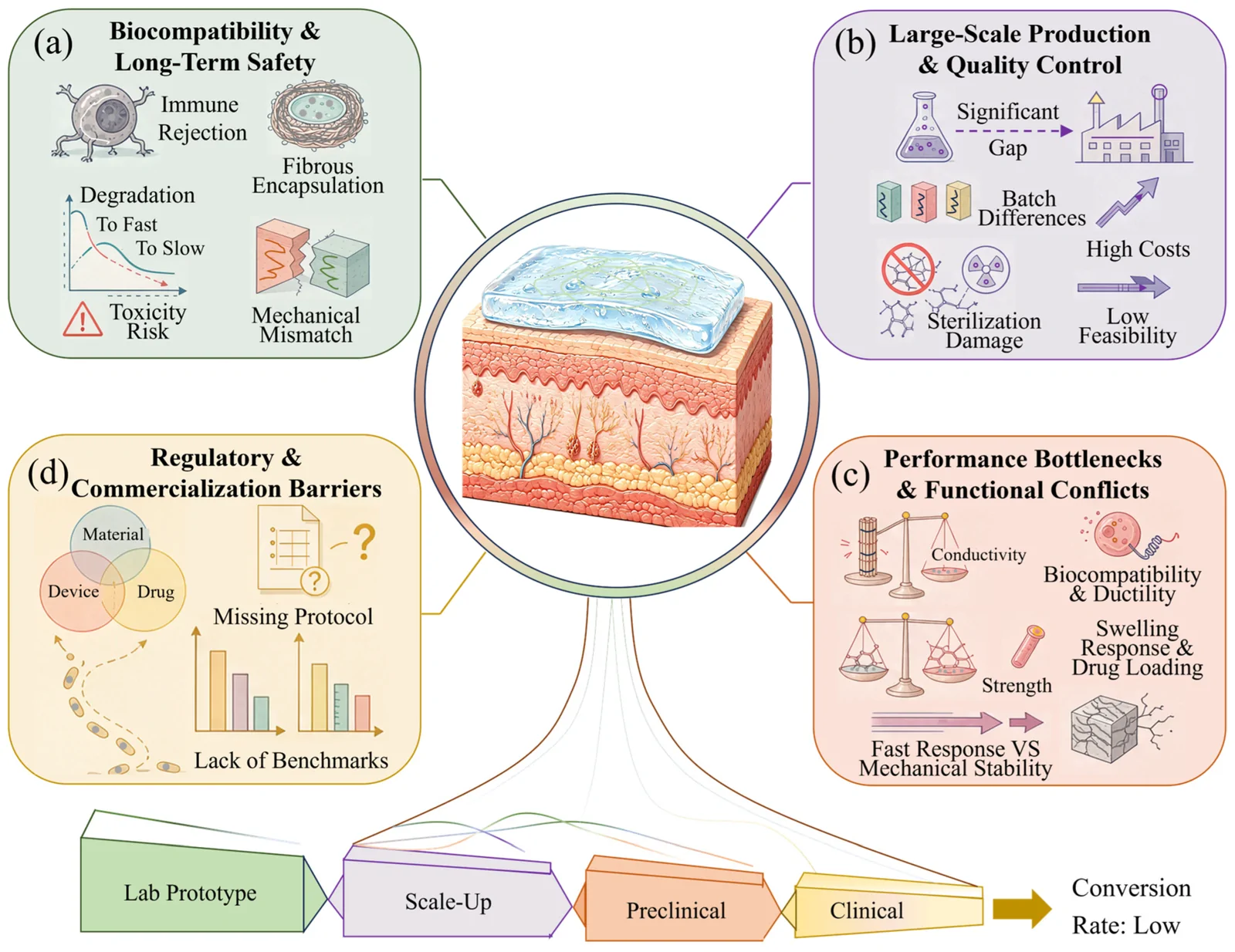
Multiple bottlenecks of intelligent hydrogel sensors from the laboratory to clinical application.

Despite their promising application prospects, only a small number of laboratory-developed intelligent hydrogel sensors have progressed toward clinical translation, leaving several critical bottlenecks yet to be addressed in this field [170,171], as shown in Figure 5.

First, biocompatibility and long-term safety pose critical challenges to implanted intelligent hydrogel sensors (Figure 5a). These unresolved issues primarily encompass acute and chronic immune rejection, functional isolation induced by in vivo fibrous capsule encapsulation, erratic degradation kinetics ranging from overly rapid to extremely slow degradation, and potential cytotoxicity of hydrogel degradation byproducts, as well as interfacial instability and cumulative micro-damage arising from mechanical property mismatch between hydrogel implants and native surrounding tissues [172,173,174]. These bottleneck problems severely restrict the long-term in vivo application and clinical translation of implantable devices. To address the above challenges, a series of up-to-date optimization strategies have been developed in recent studies. For immune rejection and fibrous capsule formation, surface hydrophilic modification, bioactive molecule immobilization, and macrophage phenotype regulation technologies are widely adopted to reduce foreign body reaction and inhibit excessive fibrous tissue hyperplasia, which effectively improves the in vivo immune compatibility of implanted materials [175,176]. For uncontrollable degradation performance, composite material blending, cross-linking degree regulation, and micro/nano-structure design can effectively modulate the in situ degradation rate of materials to match tissue regeneration rhythm, realizing synchronous degradation and tissue repair [177,178]. In terms of degradation product toxicity, raw material purification optimization and biodegradation pathway regulation strategies are applied to reduce the generation of harmful metabolites and eliminate inflammatory responses, thereby significantly improving the biosafety of degradable implants [179,180]. Furthermore, to solve the mechanical mismatch problem, gradient structure design and flexible toughening modification technologies are utilized to optimize the mechanical properties of materials, realize mechanical matching with soft/hard host tissues, and reduce interfacial friction and micro-damage during tissue movement [181,182]. In this work, we comprehensively considered the above biocompatibility and safety optimization principles to design and optimize the target biomaterial system, aiming to achieve excellent in vivo long-term service performance.

Second, large-scale production and quality control remain formidable translational barriers (Figure 5b). A substantial performance and consistency gap exists between laboratory prototypes and clinically applicable hydrogel products. During expanded production, hydrogel sensors frequently suffer from severe batch-to-batch variations, which are highly susceptible to fluctuations in raw material purity and fabrication parameters. Additionally, terminal sterilization remains challenging, as high-temperature treatment and ionizing radiation can structurally disrupt hydrogel networks and impair their functional performance. Furthermore, high manufacturing costs and the low scalability of existing fabrication procedures greatly hinder the industrial and clinical translation of intelligent hydrogel sensors [183,184].

Third, performance bottlenecks and functional conflicts substantially restrict further advancements of intelligent hydrogel sensors (Figure 5c). Hydrogel materials commonly suffer from intrinsic property conflicts, whereby the optimization of one key performance indicator inevitably compromises another. For example, incorporating highly conductive fillers such as carbon nanotubes to enhance electrical conductivity may deteriorate the hydrogel’s biocompatibility and mechanical ductility. Likewise, densely crosslinked networks designed for high mechanical strength tend to hinder swelling responsiveness and reduce drug-loading capacity. Furthermore, pursuing ultrafast sensing responses often sacrifices structural mechanical stability, creating a pervasive performance dilemma [185,186].

Finally, regulatory and commercialization barriers constitute another critical bottleneck for clinical translation (Figure 5d). Intelligent hydrogel sensors represent sophisticated integrated systems that span materials engineering, electronic devices, and pharmaceutical functionalities, leading to ambiguous product classification and uncertain regulatory approval workflows. Moreover, the absence of unified performance testing standards and evaluation benchmarks not only impedes fair and quantitative performance comparison across different studies but also increases the difficulty and cycle of regulatory review, further hindering industrial transformation and large-scale commercialization [187,188].

### 5.2. Future Development Direction

**Figure 6:**
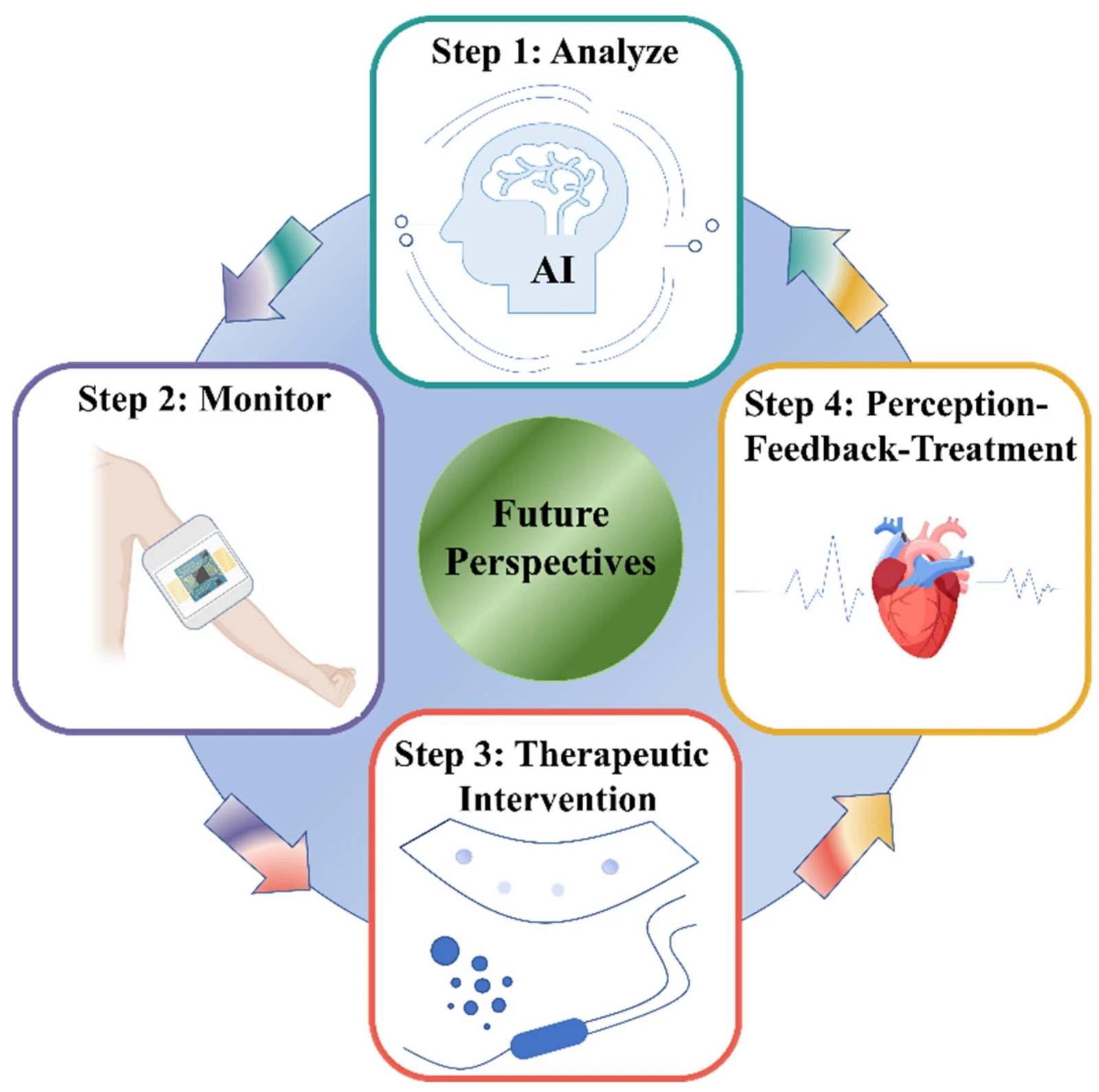
Future development direction of intelligent hydrogel sensors.

Future-generation intelligent hydrogels are evolving beyond single-response and single-function modes, pursuing ultimate performance limits and synergistic integration of multiple intelligent functionalities, as shown in Figure 6.

Future technological breakthroughs will originate from next-generation “super-materials” empowered by artificial intelligence (AI) and biomimetic design. Traditional material development relying on trial-and-error screening will be gradually replaced by data-driven predictive design and machine learning-based optimization, enabling the reverse engineering of hydrogels with precisely customized, targeted properties. Additionally, bionic strategies offer abundant innovative design inspiration. For example, mimicking the structural characteristics of human intervertebral discs enables the construction of hydrogels that simultaneously achieve high mechanical strength and high water retention capacity, while bioinspired “molecular adhesives” can be engineered to specifically capture endogenous growth factors. Beyond passive responsiveness, future hydrogel materials will achieve multifunctional synergy and programmable logical control. Such systems can execute Boolean logic operations (e.g., AND gates) in response to multiple stimuli, including pH variation, enzymatic activity, and reactive oxygen species (ROS), thereby accurately distinguishing complex pathological microenvironments.

System evolution is advancing toward intelligent biointerfaces, moving beyond isolated material units to highly integrated functional systems. Intelligent hydrogels will serve as core components embedded within sophisticated interdisciplinary platforms. First, they enable the construction of fully autonomous closed-loop theranostic systems that integrate real-time sensing, power supply, data analysis, and therapeutic feedback. Second, as ideal interfacial biomaterials, hydrogels can be seamlessly combined with flexible electronics, organ-on-a-chip platforms, and soft robotics to fabricate high-fidelity brain–computer interfaces, physiological simulation models, and biohybrid devices.

Ultimately, future research should prioritize clinical translation to bridge the “valley of death” between laboratory research and bedside application, which requires the adoption of a clinical need-driven reverse innovation paradigm. It is essential to develop low-cost, eco-friendly, and scalable manufacturing strategies, such as continuous-flow chemistry, 3D/4D bioprinting, and enzyme-catalyzed synthesis. Furthermore, advancing regulatory science is indispensable. Implementing the “quality by design” principle at the early research and development stage and collaborating with regulatory institutions can facilitate the establishment of standardized safety and efficacy evaluation criteria, thereby accelerating the clinical transformation of hydrogel theranostic platforms.

## 6. Conclusions and Prospects

Through rational biomimetic design, intelligent hydrogel sensors integrate tissue-matched mechanical compliance, tunable ionic and electronic conductivity, and multi-stimulus responsiveness. Such unique features enable the construction of a life-like biointerface that allows seamless integration with native biological systems, representing a paradigm shift in biomedical engineering toward high-fidelity physiological monitoring and precise closed-loop therapy. Accordingly, these hydrogel platforms exhibit great application potential in wearable health surveillance, implantable deep-tissue sensing, and integrated theranostic systems.

Nevertheless, translating laboratory-based novel hydrogel technologies into clinically reliable tools remains hindered by several core challenges, including long-term in vivo stability, heterogeneous functional integration, scalable and reproducible manufacturing, and standardized regulatory approval pathways. Addressing these bottlenecks requires in-depth interdisciplinary collaboration among materials scientists, bioengineers, clinicians, and regulatory professionals.

Looking ahead, once these translational bottlenecks are addressed, unobtrusive “intelligent health guardians” will come to fruition. These refer to highly miniaturized, bio-integrated, and self-powered implantable and wearable hydrogel systems. Such systems are capable of enabling continuous, personalized dynamic health monitoring and triggering autonomous, on-demand therapeutic interventions, fundamentally driving the global medical paradigm shift from passive, generalized reactive medicine toward proactive, individualized predictive and preventive medicine. Despite the remaining challenges along the translational journey, the ultimate vision of developing life-integrated functional materials for long-term health maintenance and improvement remains inspiring and scientifically and clinically significant.

## Figures and Tables

**Table 1:** Functional roles of hydrogels in various devices and definition of the core research focus of this review.

Hierarchy	Definition	The Role of the Hydrogel	Does It Fall Within the Core Scope of This Review?
Sensing technology based on hydrogels	Refers to all sensors that use hydrogels	Passive substrate, sample pad, immobilized coating	No (only as a background)
Stimuli-responsive hydrogel	The stimulus is deformed or transformed, but no signal is output	Sensing element	Part (precursor)
Smart hydrogel sensor	Stimuli-responsive hydrogel combined with a signal transduction unit can output quantifiable signals	Perception + Signal Conversion	Yes (core)
Closed-loop diagnosis and treatment system	Intelligent sensors + treatment module, achieving “perception–feedback–treatment”	Perception + Signal Conversion + Execution of Treatment	Yes (cutting-edge)

**Table 2:** Summary of typical crosslinking strategies for hydrogel biosensors.

Crosslinking Type	Mechanism	Examples	Advantages	Limitations	Refs.
Physical Crosslinking	Reversible non-covalent interactions (hydrogen bonds, ionic bonds, hydrophobic interactions, host–guest recognition)	PVA, chitosan, sodium alginate, cyclodextrin-based polymers	Mild fabrication, good biocompatibility, injectability, self-healing and stimulus-responsive performance, suitable for minimally invasive sensing	Low mechanical strength, poor physiological stability, prone to swelling and network dissociation, not applicable for long-term sensing	[54,55,56]
Chemical Crosslinking	Irreversible covalent crosslinking of polymer chains via chemical crosslinkers	PEGDA, acrylamide, GelMA (crosslinked by MBA/APS)	High mechanical strength, stable 3D network, excellent long-term structural integrity and stable sensing output under mechanical load	Irreversible network, no self-healing ability, poor flexibility and environmental adaptability, susceptible to permanent damage	[60,61,62]
Dynamic Covalent Crosslinking	Reversible dynamic covalent bonding (Schiff base, Diels–Alder reaction, borate ester coordination)	Aldehyde-modified chitosan, phenylboronic acid-functionalized polymers, furan/maleimide modified hydrogels	Balanced mechanical stability and dynamic reversibility, good fatigue resistance and environmental adaptability for complex biological sensing	Complicated preparation procedure; dynamic bonds are susceptible to pH and ionic interference, requiring structural optimization	[65,66,67]

**Table 3:** Comparison of representative intelligent hydrogel sensors for physical signal monitoring and chemical/biological marker detection.

Sensing Type	Hydrogel Composition	Sensing Mechanism	Target Signal/Analyte	Sensitivity	Response Time	Operating Range	Stability	Demonstrated Application	Refs.
Resistance/capacitance sensing	Graphene/conductive polymer hydrogel	Deformation-induced conductive network variation leads to resistance/capacitance change	Strain, pressure, pulse	Gauge factor ~10–500; strain detection down to ~0.1%	~50–200 ms	0.1–500% strain	>1000 stretching cycles	Electronic skin, pulse/respiration monitoring, silent speech recognition	[123,124,125,126]
Piezoelectric sensing	PVDF, ZnO nanowires/hydrogel	Mechanical stress induces piezoelectric polarization to generate electrical signals	Mechanical stress, pressure	Output voltage ~ mV range; pressure sensitivity ~ mV·kPa^−1^	<10 ms	Wide frequency range (0.1–100 Hz)	Long-term, self-powered	Implantable energy harvesting, intracardiac pressure monitoring	[130,131]
Acoustic/mechanical sensing	MXene/PVA hydrogel	Ultrasonic excitation-induced electromagnetic coupling response	Ultrasonic waves, mechanical vibration	Field intensity tunable; high spatial precision	Real-time (ultrasonic response)	Adjustable alternating electromagnetic field	Biodegradable and biocompatible	Glioblastoma treatment device	[136]
Electrochemical sensing	Conductive hydrogel+ enzymes/antibodies	Biochemical reaction induces current/potential/impedance variation for quantitative detection	Glucose, dopamine, serotonin	Glucose: μM-mM; dopamine: nM-μM	~1–10 s	Physiological concentration range	Days to weeks (continuous)	Continuous glucose monitoring; brain neurochemical detection	[141,142,143,144,145,146]
Optical sensing	Hydrogel + fluorescent probes/SERS nanomaterials	Biomolecular binding triggers specific optical signal amplification	Biomarkers, exosomes, antigens	LOD: pg·mL^−1^-ng·mL^−1^ (SERS); nM-pM (fluorescence)	~1–30 min	Wide dynamic range	Stable under storage	COVID-19 antigen detection; cancer exosome detection	[150,151,152]
Affinity sensing	Hydrogel-modified SPR interface	Specific molecular recognition changes interfacial refractive index for signal monitoring	Antigens, nucleic acids, drug targets	Label-free; affinity range nM-pM	Real-time (seconds)	Wide concentration range	Reusable, stable	Drug target screening; biomolecular interaction studies	[156,157]

**Table 4:** Comparison of different closed-loop systems.

Perceived Target	Perceived Mechanism	Decision/Feedback Component	Treatment Executor	Response Time	Degree of In Vivo Validation	Refs.
Blood glucose	Glucose oxidase catalyzes the production of H_2_O_2_, which triggers the cross-linking breakage of phenylboronic acid	The degradation of the hydrogel leads to the release of insulin	Insulin	From several minutes to several hours	Subcutaneous implantation in mice, effectively lowering blood sugar	[166]
Reactive Oxygen Species (ROS)	ROS oxidizes the sulfur ether bond, and the hydrogel disintegrates	Disrupts the release of antioxidant enzymes in the package	Catalase/Superoxide Dismutase	Minute-level	Rat model of myocardial ischemia–reperfusion injury	[167]
Local pH (tumor microenvironment)	The pH-sensitive bidentate bond breaks, and the hydrogel disintegrates	Disintegration to release chemotherapy drugs	Doxorubicin	Several hours	Subcutaneous mouse model of breast cancer	[168]
Inflammatory factors (such as TNF-α)	Adaptor–antigen binding leads to hydrogel swelling	Drug release through swelling and compression micro-needle arrays	Anti-TNF-α antibody	Several hours	Mouse arthritis model, intra-articular injection	[169]

## Data Availability

No new data were created or analyzed in this study. Data sharing is not applicable to this article.

## References

[1] Gianfredi V., Nucci D., Pennisi F., Maggi S., Veronese N., Soysal P. (2025). Aging, longevity, and healthy aging: The public health approach. Aging Clin. Exp. Res..

[2] Behr L.C., Simm A., Kluttig A., Grosskopf A. (2023). 60 years of healthy aging: On definitions, biomarkers, scores and challenges. Ageing Res. Rev..

[3] Katherine R.M., Isabel S.M. (2025). Physical activity and healthy ageing in the era of chronic conditions. Br. J. Sports Med..

[4] Hartl D., de Luca V., Kostikova A., Laramie J., Kennedy S., Ferrero E., Siegel R., Fink M., Ahmed S., Millholland J. (2021). Translational precision medicine: An industry perspective. J. Transl. Med..

[5] Yuan J.Y., Zhang S., Han D., Dong X.Y. (2026). Agrin at the crossroads of aging: A pleiotropic regulator in age-related diseases. Pharmacol. Res..

[6] Teng Y.J., Yang J.N., Li T.M., Yin Z.H., An Q., Shi Z.Q., Jia D.G., Lv Q., Ramakrishna S., Shi J. (2026). The cutting-edge advancements in biomaterials under the guidance of intelligence and bionics. Regen. Biomater..

[7] Loupy A., Sablik M., Khush K., Reese P.P. (2025). Advancing patient monitoring, diagnostics, and treatment strategies for transplant precision medicine. Lancet.

[8] Lee Y.A., Lee J.Y., Kim J., Lee H., Park S., Sunwoo S.-H., Cha G.D., Kim D.-H. (2026). Chemically anchored metal-hydrogel bilayers for ultrasoft and metallic biointerfaces. Nanoscale Horiz..

[9] Ahamad S., Fatima S., Singh K., Ravindranath S.S., Devi S.G., Ali A., Kumar A., Manik G. (2026). Biopolymer-based stimuli-responsive and self-healing conductive hydrogels for next-generation bioelectronics interfaces. ACS Appl. Polym. Mater..

[10] Araújo A.A.C., Gardim L., Salma J., Stephen T., dos Santos S.S., Silva Í.R., de Godoy S., Mendes I.A.C. (2024). Advancing nursing education through wearable electronic devices: A scoping review. Nurse Educ. Pract..

[11] Chen X., Kim D.-H., Lu N. (2024). Introduction: Wearable Devices. Chem. Rev..

[12] Pozdin V.A., Dieffenderfer J. (2022). Towards Wearable Health Monitoring Devices. Biosensors.

[13] Maria C.T., Francesco N., Nehar C., Żaneta G., Pasquale P., Wojciech Ś., Marco C. (2026). Microfluidic-mixer-assisted 3D printing of functionally graded multimaterial hydrogels for engineering complex tissue interfaces. Mater. Adv..

[14] Karako K. (2024). Integration of wearable devices and deep learning: New possibilities for health management and disease prevention. Biosci. Trends.

[15] Wu P., An X.F., Zheng B.H., Wang W.Q., Wang K.X., Liu H.B., Yao Y., Zhou H.W. (2026). In situ generated bubble-mediated porous ionically conductive hydrogels for hydrogel-based electronics. Mater. Horiz..

[16] Baniasadi H. (2025). State-of-the-art in natural hydrogel-based wound dressings: Design, functionalization, and fabrication approaches. Adv. Colloid Interface Sci..

[17] Zou X.R., Zheng Y.J., Zhang X.F., Ma S., Jia X.Y., Tang E.M., Xiao Z.L., Zhang L., Hou Y.L. (2026). Interfacial insights into hydrogel electrolytes in flexible aqueous zinc batteries: From materials to devices. Energy Environ. Sci..

[18] Herrmann A., Haag R., Schedler U. (2021). Hydrogels and Their Role in Biosensing Applications. Adv. Healthc. Mater..

[19] Drozd M., Karoń S., Malinowska E. (2021). Recent Advancements in Receptor Layer Engineering for Applications in SPR-Based Immunodiagnostics. Sensors.

[20] Mo Y., Zhou T., Li W., Niu Y., Sheu C. (2025). Advances in Nanohybrid Hydrogels for Wound Healing: From Functional Mechanisms to Translational Prospects. Gels.

[21] Wang K., Zhang J., Li H., Wu J., Wan Q., Chen T., Liu W., Peng H., Zhang H., Luo Y. (2024). Smart Hydrogel Sensors for Health Monitoring and Early Warning. Adv. Sens. Res..

[22] Li Y., Tan S., Zhang X., Li Z., Cai J., Liu Y. (2025). Design Strategies and Emerging Applications of Conductive Hydrogels in Wearable Sensing. Gels.

[23] Qu J., Xie K., Chen S., He X., Wang Y., Chamberlin M., Zhao X., Zhu G., Xu C., Shi P. (2024). Multifunctional hydrogel electronics for closed-loop antiepileptic treatment. Sci. Adv..

[24] Niu Y., Zhao Z., Yang L., Lv D., Sun R., Zhang T., Li Y., Bao Q., Zhang M., Wang L. (2025). Towards Intelligent Wound Care: Hydrogel-Based Wearable Monitoring and Therapeutic Platforms. Polymers.

[25] Chung T., Choi J., Enomoto T., Park S., Kim S., Kim Y.S. (2025). Self-Regul. Hydrogel Actuators. Chem. Rev..

[26] Liu R., Liu Y., Fu S., Cheng Y., Jin K., Ma J., Wan Y., Tian Y. (2024). Humidity Adaptive Antifreeze Hydrogel Sensor for Intelligent Control and Human-Computer Interaction. Small.

[27] She Y., Liu H., Yuan H., Li Y., Liu X., Liu R., Wang M., Wang T., Wang L., Liu M. (2025). Artificial Intelligence-Assisted Conductive Hydrogel Dressings for Refractory Wounds Monitoring. Nano-Micro Lett..

[28] Wang H., Song L., Chen C., Li K., Wei M., Wang M., Li N., Yang M., Yuan W. (2025). Mechanically Adaptive, Self-Adhesive, Conductive, Cross-Linked Interpenetrating Zwitterionic Hydrogel Sensor for Simultaneous Wound Healing and Monitoring. ACS Appl. Mater. Interfaces.

[29] Kędzierska M., Sala K., Bańkosz M., Grzela K., Potemski P., Miernik K., Tyliszczak B. (2024). Enhanced Hydrogel Materials: Incorporating Vitamin C and Plant Extracts for Biomedical Applications. Molecules.

[30] Polla Ravi S., Shamiya Y., Chakraborty A., Elias C., Paul A. (2021). Biomaterials, biological molecules, and polymers in developing vaccines. Trends Pharmacol. Sci..

[31] Dargaville B.L., Hutmacher D.W. (2022). Water as the often neglected medium at the interface between materials and biology. Nat. Commun..

[32] Acciaretti F., Vesentini S., Cipolla L. (2022). Fabrication Strategies Towards Hydrogels for Biomedical Application: Chemical and Mechanical Insights. Chem.-Asian J..

[33] Xu F., Dawson C., Lamb M., Mueller E., Stefanek E., Akbari M., Hoare T. (2022). Hydrogels for Tissue Engineering: Addressing Key Design Needs Toward Clinical Translation. Front. Bioeng. Biotechnol..

[34] Wang Z., Wei H., Huang Y., Wei Y., Chen J. (2023). Naturally sourced hydrogels: Emerging fundamental materials for next-generation healthcare sensing. Chem. Soc. Rev..

[35] Cai C., Li W., Zhang X., Cheng B., Chen S., Zhang Y. (2025). Natural Polymer–Based Hydrogel Dressings for Wound Healing. Adv. Wound Care.

[36] Afrin S., Siddiqua Prova O., Qureshi A.T., Ishaq M.W., Callmann C.E., Rizwan M. (2026). Injectable and self-healing fucoidan hydrogel: A natural anti-inflammatory biomaterial. Biomaterials.

[37] Ho T.-C., Chang C.-C., Chan H.-P., Chung T.-W., Shu C.-W., Chuang K.-P., Duh T.-H., Yang M.-H., Tyan Y.-C. (2022). Hydrogels: Properties and Applications in Biomedicine. Molecules.

[38] Farshidfar N., Iravani S., Varma R.S. (2023). Alginate-Based Biomaterials in Tissue Engineering and Regenerative Medicine. Mar. Drugs.

[39] Zhang R., Zhao C., Liu C., Gluchman M., Yang H., Li W.-Y. (2025). Hydrogel sealants in hemostasis: A review of natural, synthetic, and composite biological macromolecules. Int. J. Biol. Macromol..

[40] Zhang Q., Wang X., Kang Y., Yao C., Li X., Li L. (2022). Conjugated Molecule-Assisted Supramolecular Hydrogel with Enhanced Antibacterial and Antibiofouling Properties. ACS Appl. Bio Mater..

[41] Chen Z.-Y., Zhou R.-B., Wang R.-D., Su S.-L., Zhou F. (2023). Dual-crosslinked network of polyacrylamide-carboxymethylcellulose hydrogel promotes osteogenic differentiation in vitro. Int. J. Biol. Macromol..

[42] Li J., Wang L., Jiang G., Wan Y., Wang J., Li Y., Pi F. (2024). Luminescent carbon dots-rooted polysaccharide crosslinked hydrogel adsorbent for sensitive determination and efficient removal of Cu2+. Food Chem..

[43] Xu Z., Liu G., Huang J., Wu J. (2022). Novel Glucose-Responsive Antioxidant Hybrid Hydrogel for Enhanced Diabetic Wound Repair. ACS Appl. Mater. Interfaces.

[44] Nakahata M., Tominaga N., Saito K., Nishiyama K., Tanino Y., Saiki K., Kojima M., Sakai S. (2022). A Bio-synthetic Hybrid Hydrogel Formed under Physiological Conditions Consisting of Mucin and a Synthetic Polymer Carrying Boronic Acid. Macromol. Biosci..

[45] Cai Y., Xin L., Sun P., Li H., Liu C., Fang L. (2023). Temperature-sensitive multifunctional intelligent responsive hydrogel based on carboxymethyl agarose and N-isopropylacrylamide: Controlled drug release and accelerated wound healing. Carbohydr. Polym..

[46] Wu M., Liu H., Li D., Zhu Y., Wu P., Chen Z., Chen F., Chen Y., Deng Z., Cai L. (2023). Smart-Responsive Multifunctional Therapeutic System for Improved Regenerative Microenvironment and Accelerated Bone Regeneration via Mild Photothermal Therapy. Adv. Sci..

[47] Chen Y., Wang X., Tao S., Wang Q., Ma P.-Q., Li Z.-B., Wu Y.-L., Li D.-W. (2023). Research advances in smart responsive hydrogel dressings with potential clinical diabetic wound healing properties. Mil. Med. Res..

[48] Tian B., Liu J. (2023). Smart stimuli-responsive chitosan hydrogel for drug delivery: A review. Int. J. Biol. Macromol..

[49] Xu Q., Zhang B., Li H., Liu C., Bi S., Yang Z., Liu Y. (2025). Application and progress of intelligent responsive hydrogels in articular cartilage injury repair. Chin. J. Reparative Reconstr. Surg..

[50] Sarmah D., Rather M.A., Sarkar A., Mandal M., Sankaranarayanan K., Karak N. (2023). Self-cross-linked starch/chitosan hydrogel as a biocompatible vehicle for controlled release of drug. Int. J. Biol. Macromol..

[51] Sarmah D., Karak N. (2022). Physically cross-linked starch/hydrophobically-associated poly(acrylamide) self-healing mechanically strong hydrogel. Carbohydr. Polym..

[52] Garg A., Agrawal R., Singh Chauhan C., Deshmukh R. (2024). In-situ gel: A smart carrier for drug delivery. Int. J. Pharm..

[53] De Maeseneer T., Van Damme L., Aktan M.K., Braem A., Moldenaers P., Van Vlierberghe S., Cardinaels R. (2024). Powdered Cross-Linked Gelatin Methacryloyl as an Injectable Hydrogel for Adipose Tissue Engineering. Gels.

[54] Thirupathi K., Raorane C.J., Ramkumar V., Ulagesan S., Santhamoorthy M., Raj V., Krishnakumar G.S., Phan T.T.V., Kim S.-C. (2022). Update on Chitosan-Based Hydrogels: Preparation, Characterization, and Its Antimicrobial and Antibiofilm Applications. Gels.

[55] Bertsch P., Diba M., Mooney D.J., Leeuwenburgh S.C.G. (2022). Self-Healing Injectable Hydrogels for Tissue Regeneration. Chem. Rev..

[56] Fan X., Zhao L., Ling Q., Gu H. (2022). Tough, Self-Adhesive, Antibacterial, and Recyclable Supramolecular Double Network Flexible Hydrogel Sensor Based on PVA/Chitosan/Cyclodextrin. Ind. Eng. Chem. Res..

[57] Habib M., Berthalon S., Leclercq L., Tourrette A., Sharkawi T., Blanquer S. (2024). Dual Cross-Linked Stimuli-Responsive Alginate-Based Hydrogels. Biomacromolecules.

[58] Bahou C., Spears R.J., Ramírez Rosales A.M., Rochet L.N.C., Barber L.J., Stankevich K.S., Miranda J.F., Butcher T.C., Kerrigan A.M., Lazarov V.K. (2023). Hydrogel Cross-Linking via Thiol-Reactive Pyridazinediones. Biomacromolecules.

[59] Kushwaha R., Dey S., Gupta K., Mandal B.B., Das D. (2024). Secondary Chemical Cross-Linking to Improve Mechanical Properties in a Multifaceted Biocompatible Strain Sensor. ACS Appl. Mater. Interfaces.

[60] Yue K., Trujillo-de Santiago G., Alvarez M.M., Tamayol A., Annabi N., Khademhosseini A. (2015). Synthesis, properties, and biomedical applications of gelatin methacryloyl (GelMA) hydrogels. Biomaterials.

[61] Choi J.R., Yong K.W., Choi J.Y., Cowie A.C. (2019). Recent Advances in Photo-Crosslinkable Hydrogels for Biomedical Applications. BioTechniques.

[62] Gong W., Kim J., Kim C., Chang H., Ahn Y., Schaffer D.V., Baek J. (2025). Hydrogel fabrication techniques for advanced artificial sensory systems. Int. J. Extrem. Manuf..

[63] Lee Y.-M., Lu Z.-W., Wu Y.-C., Liao Y.-J., Kuo C.-Y. (2024). An injectable, chitosan-based hydrogel prepared by Schiff base reaction for anti-bacterial and sustained release applications. Int. J. Biol. Macromol..

[64] Ruiz-Pardo C., Silva-Gutiérrez L., Lizardi-Mendoza J., López-Franco Y., Peniche-Covas C., Argüelles-Monal W. (2022). Chitosan Hydrogels Based on the Diels-Alder Click Reaction: Rheological and Kinetic Study. Polymers.

[65] Choi C., Seo D., Kim T., Park J., Yu D., Yu S., Shin J., Yuk S.A., Sung D., Kim H. (2026). Engineering Smart Hydrogels Through Dynamic Polymer Networks for Controlled Drug Delivery. Gels.

[66] Joshi S., Baker M.B., Schauenburg D. (2026). Chemical Strategies for Multistimuli Responsive Dynamic Covalent Materials in Regenerative Medicine. Adv. Healthc. Mater..

[67] Wang M., Zhang Z., Li G., Jing A. (2023). Room-Temperature Self-Healing Conductive Elastomers for Modular Assembly as a Microfluidic Electrochemical Biosensing Platform for the Detection of Colorectal Cancer Exosomes. Micromachines.

[68] Wilson A.D. (2023). Developments of Recent Applications for Early Diagnosis of Diseases Using Electronic-Nose and Other VOC-Detection Devices. Sensors.

[69] Chen F., Zhuang Q., Ding Y., Zhang C., Song X., Chen Z., Zhang Y., Mei Q., Zhao X., Huang Q. (2023). Wet-Adaptive Electronic Skin. Adv. Mater..

[70] Yu A., Zhu M., Chen C., Li Y., Cui H., Liu S., Zhao Q. (2023). Implantable Flexible Sensors for Health Monitoring. Adv. Healthc. Mater..

[71] Bierman-Duquette R.D., Safarians G., Huang J., Rajput B., Chen J.Y., Wang Z.Z., Seidlits S.K. (2021). Engineering Tissues of the Central Nervous System: Interfacing Conductive Biomaterials with Neural Stem/Progenitor Cells. Adv. Healthc. Mater..

[72] Bari G.A.K.M.R., Jeong J.-H., Barai H.R. (2024). Conductive Gels for Energy Storage, Conversion, and Generation: Materials Design Strategies, Properties, and Applications. Materials.

[73] Liu D., Wang X., Gao C., Zhang Z., Wang Q., Pei Y., Wang H., Tang Y., Li K., Yu Y. (2024). Biodegradable Piezoelectric-Conductive Integrated Hydrogel Scaffold for Repair of Osteochondral Defects. Adv. Mater..

[74] Yang H., Zheng H., Duan Y., Xu T., Xie H., Du H., Si C. (2023). Nanocellulose-graphene composites: Preparation and applications in flexible electronics. Int. J. Biol. Macromol..

[75] Manjunatha J.G., Uslu B. (2022). Carbon-Based Composite Materials for Electrodes. Materials.

[76] Kwon D.-H., Jeong J., Lee Y., Park J.-K., Lee S., Bae J.-H., Kim H. (2021). Carbon Nanotube-Polymer Hybrid Nanocomposite Electrodes for Porous Polydimethylsiloxane Sponge-Based Flexible Triboelectric Nanogenerators. J. Nanosci. Nanotechnol..

[77] Joshi R., Ravindran K.V., Lahiri I. (2025). Graphene-based materials and electrochemical biosensors: An overview. J. Phys. Condens. Matter.

[78] Wang H., Zhao Z., Liu P., Guo X. (2022). Laser-Induced Graphene Based Flexible Electronic Devices. Biosensors.

[79] Tang H., Li Y., Liao S., Liu H., Qiao Y., Zhou J. (2024). Multifunctional Conductive Hydrogel Interface for Bioelectronic Recording and Stimulation. Adv. Healthc. Mater..

[80] Gao Y., Dai C., Zhang M., Zhang J., Yin L., Li W., Zhang K., Yang Y., Zhao Y. (2023). Biomimetic Silk Fibroin Hydrogel for Enhanced Peripheral Nerve Regeneration: Synergistic Effects of Graphene Oxide and Fibroblast Exosome. Adv. Funct. Mater..

[81] He X., Wang R., Liu X., Peng R., Zhou B., Wang L., Wei X., Wang S., Bai J., Feng Q. (2025). Customizable Silk Fibroin-Based Hydrogel Fibrous Scaffold for On-Demand Multifaceted Tissue Repair. ACS Nano.

[82] Escobar A., Carvalho M.R., Silva T.H., Reis R.L., Oliveira J.M. (2023). Longitudinally aligned inner-patterned silk fibroin conduits for peripheral nerve regeneration. Vitr. Models.

[83] Jordan R.S., Frye J., Hernandez V., Prado I., Giglio A., Abbasizadeh N., Flores-Martinez M., Shirzad K., Xu B., Hill I.M. (2021). 3D printed architected conducting polymer hydrogels. J. Mater. Chem. B.

[84] Le C.V., Yoon H. (2024). Advances in the Use of Conducting Polymers for Healthcare Monitoring. Int. J. Mol. Sci..

[85] Keirouz A., Mustafa Y.L., Turner J.G., Lay E., Jungwirth U., Marken F., Leese H.S. (2023). Conductive Polymer-Coated 3D Printed Microneedles: Biocompatible Platforms for Minimally Invasive Biosensing Interfaces. Small.

[86] Kougkolos G., Golzio M., Laudebat L., Valdez-Nava Z., Flahaut E. (2023). Hydrogels with electrically conductive nanomaterials for biomedical applications. J. Mater. Chem. B.

[87] Heredia Rivera U., Kadian S., Nejati S., White J., Sedaghat S., Mutlu Z., Rahimi R. (2022). Printed Low-Cost PEDOT: PSS/PVA Polymer Composite for Radiation Sterilization Monitoring. ACS Sens..

[88] Shakibania S., Patel T., Turczyn R., Biggs M.J.P., Krukiewicz K. (2025). Hybrid conducting polymer films promote neural outgrowth and neural-electrode integration in vitro. Bioelectrochemistry.

[89] Zhong H., Shan W., Liang L., Jiang X., Wu L. (2024). High stretchable and self-adhesive multifunctional hydrogel for wearable and flexible sensors. Heliyon.

[90] Li Y., Zhang X., Tan S., Li Z., Sun J., Li Y., Xie Z., Li Z., Han F., Liu Y. (2025). Design Strategies of PEDOT: PSS-Based Conductive Hydrogels and Their Applications in Health Monitoring. Polymers.

[91] Zhang W., Li Z., Zhang Q., Zheng S., Zhang Z., Chen S., Wang Z., Zhang D. (2024). Ionic conducting hydrogels as biomedical materials: Classification, design strategies, and skin tissue engineering applications. J. Biomater. Sci. Polym. Ed..

[92] Li M., Wang S., Li Y., Meng X., Wei Y., Wang Y., Chen Y., Xiao Y., Cheng Y. (2024). An Integrated All-Natural Conductive Supramolecular Hydrogel Wearable Biosensor with Enhanced Biocompatibility and Antibacterial Properties. ACS Appl. Mater. Interfaces.

[93] Sun J., Wu X., Xiao J., Zhang Y., Ding J., Jiang J., Chen Z., Liu X., Wei D., Zhou L. (2023). Hydrogel-Integrated Multimodal Response as a Wearable and Implantable Bidirectional Interface for Biosensor and Therapeutic Electrostimulation. ACS Appl. Mater. Interfaces.

[94] Ariaeenejad S., Motamedi E., Salimi M., Tayi F., Zahouily M. (2026). Enzyme immobilization on hydrogels: An overview on methods, interactions, and divers applications. Int. J. Biol. Macromol..

[95] Tavakoli J., Tang Y. (2017). Hydrogel Based Sensors for Biomedical Applications: An Updated Review. Polymers.

[96] Zustiak S.P., Wei Y., Leach J.B. (2013). Protein-Hydrogel Interactions in Tissue Engineering: Mechanisms and Applications. Tissue Eng. Part B Rev..

[97] Hu M., Tang Y., He X., Liu K., Qin L., Wang X., Wang Q. (2025). Enzyme-Integrated Hydrogels for Advanced Biological Applications. Polym. Sci. Technol..

[98] Sun G., Wei X., Zhang D., Huang L., Liu H., Fang H. (2023). Immobilization of Enzyme Electrochemical Biosensors and Their Application to Food Bioprocess Monitoring. Biosensors.

[99] Kalyana Sundaram S.d., Hossain M.M., Rezki M., Ariga K., Tsujimura S. (2023). Enzyme Cascade Electrode Reactions with Nanomaterials and Their Applicability towards Biosensor and Biofuel Cells. Biosensors.

[100] Patil P.D., Salokhe S., Karvekar A., Suryavanshi P., Phirke A.N., Tiwari M.S., Nadar S.S. (2023). Microfluidic based continuous enzyme immobilization: A comprehensive review. Int. J. Biol. Macromol..

[101] Wang X., Qin Z., Zhang F., Li C., Yuan X., Yang J., Yang H. (2024). Label-free electrochemical biosensor based on dual amplification of gold nanoparticles and polycaprolactones for CEA detection. Talanta.

[102] Li Y., Wang X., Wang X., Qin Z., Li C., Yang J., Cao M. (2025). Electrochemical biosensor based on composite of gold nanoparticle/reduced-graphene oxide/graphitic carbon nitride and a caprolactone polymer for highly sensitive detection of CEA. Bioelectrochemistry.

[103] Dong H., Liu X., Gan L., Fan D., Sun X., Zhang Z., Wu P. (2023). Nucleic acid aptamer based biosensors and their application in thrombin analysis. Bioanalysis.

[104] Crnoglavac Popović M., Stanković V., Stanković D., Prodanović R. (2025). Protein, Nucleic Acid, and Nanomaterial Engineering for Biosensors and Monitoring. Biosensors.

[105] Janghorban M., Aradanas I., Kazemi S., Ngaju P., Pandey R. (2022). Recent Advances, Opportunities, and Challenges in Developing Nucleic Acid Integrated Wearable Biosensors for Expanding the Capabilities of Wearable Technologies in Health Monitoring. Biosensors.

[106] Lam S.Y., Lau H.L., Kwok C.K. (2022). Capture-SELEX: Selection Strategy, Aptamer Identification, and Biosensing Application. Biosensors.

[107] Gan Z., Roslan M.A.M., Abd Shukor M.Y., Halim M., Yasid N.A., Abdullah J., Md Yasin I.S., Wasoh H. (2022). Advances in Aptamer-Based Biosensors and Cell-Internalizing SELEX Technology for Diagnostic and Therapeutic Application. Biosensors.

[108] Jin S., Newton M.A.A., Cheng H., Zhang Q., Gao W., Zheng Y., Lu Z., Dai Z., Zhu J. (2023). Progress of Hydrogel Dressings with Wound Monitoring and Treatment Functions. Gels.

[109] Zhang T., Meng Z., Yu H., Ding P., Kai T. (2025). An Intelligent and Conductive Hydrogel with Multiresponsive and ROS Scavenging Properties for Infection Prevention and Anti-Inflammatory Treatment Assisted by Electrical Stimulation for Diabetic Wound. Adv. Sci..

[110] Li Z., Lu J., Ji T., Xue Y., Zhao L., Zhao K., Jia B., Wang B., Wang J., Zhang S. (2024). Self-Healing Hydrogel Bioelectronics. Adv. Mater..

[111] Shan M., Chen X., Zhang X., Zhang S., Zhang L., Chen J., Wang X., Liu X. (2024). Injectable Conductive Hydrogel with Self-Healing, Motion Monitoring, and Bacteria Theranostics for Bioelectronic Wound Dressing. Adv. Healthc. Mater..

[112] Chen J., Wang L., Xu X., Liu G., Liu H., Qiao Y., Chen J., Cao S., Cha Q., Wang T. (2022). Self-Healing Materials-Based Electronic Skin: Mechanism, Development and Applications. Gels.

[113] Fu Y., Dang X. (2025). Bio-Inspired Highly Stretchable and Ultrafast Autonomous Self-Healing Supramolecular Hydrogel for Multifunctional Durable Self-Powered Wearable Devices. Small.

[114] Chen M., Chen T., Bai J., He S., Luo M., Zeng Y., Peng W., Zhao Y., Wang J., Zhu X. (2023). A Nature-Inspired Versatile Bio-Adhesive. Adv. Healthc. Mater..

[115] Song P., Sun Q., Liang X., Li J., Shi J., Zhu Y., Du J. (2025). Reversible Adhesive Hydrogel Balancing Conflicting Needs of Strong Adhesion and_Painless Removal for Diabetic Joint Wound Healing. ACS Nano.

[116] Liu Y., Wang C., Xue J., Huang G., Zheng S., Zhao K., Huang J., Wang Y., Zhang Y., Yin T. (2022). Body Temperature Enhanced Adhesive, Antibacterial, and Recyclable Ionic Hydrogel for Epidermal Electrophysiological Monitoring. Adv. Healthc. Mater..

[117] Koo H.B., Heo E., Cho I., Kim S.H., Kang J., Chang J.-B. (2023). Human hand-inspired all-hydrogel gripper with a high load capacity formed by the split-brushing adhesion of diverse hydrogels. Mater. Horiz..

[118] Zhang X., Jiang S., Ma G., Tang H., Ma J., Liu Y., Si L., Zhang S., Yu Y., Liu Y. (2026). Bioinspired bioactive bilayer membrane: A biodegradable adhesive membrane integrating hemostasis, antibacterial, and anti-adhesion functions for visceral repair. Carbohydr. Polym..

[119] An S., Jeon E.J., Han S.Y., Jeon J., Lee M.J., Kim S., Shin M., Cho S.W. (2022). pH-Universal Catechol-Amine Chemistry for Versatile Hyaluronic Acid Bioadhesives. Small.

[120] Lin H., Wang Z., Li P., Zhang Y., Kong W., Yue X., Feng L., Chen Z., Zheng Y., Hu Z. (2026). Intelligent-responsive hydrogel synergistically mediates immune remodel-antibacterial-angiogenesis cascade for diabetic foot ulcer repair. Bioact. Mater..

[121] Han Y., Weng W., Zhang Y., Feng Q., Ma Y., Quan A., Fu X., Zhao X., Skudder-Hill L., Jiang J. (2024). Intraoperative application of intelligent, responsive, self-assembling hydrogel rectifies oxygen and energy metabolism in traumatically injured brain. Biomaterials.

[122] Li J., Gao J., Zhu K., Gao Y. (2026). Research advances in intelligent microenvironment-responsive hydrogels for myocardial infarction therapy. APL Bioeng..

[123] Tian L., Yang Z., Wang Q. (2026). Nanoconfinement-enabled GO hydrogels via Hummers’ method for real-time health monitoring. RSC Adv..

[124] Tang C., Xu M., Yi W., Zhang Z., Occhipinti E., Dong C., Ravenscroft D., Jung S.-M., Lee S., Gao S. (2024). Ultrasensitive textile strain sensors redefine wearable silent speech interfaces with high machine learning efficiency. npj Flex. Electron..

[125] Li R., Hu J., Li Y., Huang Y., Wang L., Huang M., Wang Z., Chen J., Fan Y., Chen L. (2025). Graphene-Based, Flexible, Wearable Piezoresistive Sensors with High Sensitivity for Tiny Pressure Detection. Sensors.

[126] Kong M., Zhou R., Yang M., Zhang J., Ma X., Gao T., Zhang Y., Li B., Liu M., Cui X. (2024). Mechanism and Performance Evaluation of a Strain Sensor Made from a Composite Hydrogel Containing Conductive Fibers of Thermoplastic Polyurethane and Polyvinyl Alcohol. ACS Omega.

[127] He Y., Xu X., Xiao S., Wu J., Zhou P., Chen L., Liu H. (2024). Research Progress and Application of Multimodal Flexible Sensors for Electronic Skin. ACS Sens..

[128] Bao L., Han C., Li G., Chen J., Wang W., Yang H., Huang X., Guo J., Wu H. (2023). Flexible Electronic Skin for Monitoring of Grasping State During Robotic Manipulation. Soft Robot..

[129] Yang Z., Yu X., Song Y., Hu Y., Yang Q., Xiong C., Shi Z. (2024). Flexible and ultrasensitive piezoresistive electronic skin based on chitin/sulfonated carbon nanotube films. Int. J. Biol. Macromol..

[130] Du Y., Du W., Lin D., Ai M., Li S., Zhang L. (2023). Recent Progress on Hydrogel-Based Piezoelectric Devices for Biomedical Applications. Micromachines.

[131] Chang G., Pan X., Hao Y., Du W., Wang S., Zhou Y., Yang J., He Y. (2024). PVDF/ZnO piezoelectric nanofibers designed for monitoring of internal micro-pressure. RSC Adv..

[132] Meshkinzar A., Al-Jumaily A.M. (2023). Cylindrical Piezoelectric PZT Transducers for Sensing and Actuation. Sensors.

[133] Cai X., Wang Y., Cao Y., Yang W., Xia T., Li W. (2024). Flexural-Mode Piezoelectric Resonators: Structure, Performance, and Emerging Applications in Physical Sensing Technology, Micropower Systems, and Biomedicine. Sensors.

[134] Lee E., Yoo H. (2021). Self-Powered Sensors: New Opportunities and Challenges from Two-Dimensional Nanomaterials. Molecules.

[135] Liu Z., Ma Y., Ouyang H., Shi B., Li N., Jiang D., Xie F., Qu D., Zou Y., Huang Y. (2018). Transcatheter Self-Powered Ultrasensitive Endocardial Pressure Sensor. Adv. Funct. Mater..

[136] Hu X., Qiu Z., Yang Y., Xu T., Sheng K., Lu W., Xie J., Xu B. (2024). Implantable Ultrasound-Powered MXene/PVA Hydrogel-Based Generator for Treatment of Glioblastoma. Adv. Sci..

[137] Byun E., Nam J., Shim H., Kim E., Kim A., Song S. (2020). Ultrasonic hydrogel biochemical sensor system. Proceedings of the 2020 42nd Annual International Conference of the IEEE Engineering in Medicine & Biology Society (EMBC), Montreal, QC, Canada, 20–24 July 2020.

[138] Lu Z., Wang J., He L., Song J., Yang Z., Hammad F.A. (2024). High-Performance Multidirectional Flexible Strain Sensor for Human Motion and Health Monitoring. ACS Appl. Mater. Interfaces.

[139] Tal S., Tchizick A., Shamay-Tsoory S., Dolev-Amit T., Zilcha-Mano S. (2023). Biological marker of withdrawal ruptures: Dyadic pattern of incongruence in oxytocin release. J. Couns. Psychol..

[140] Duan B., Guo T., Sun H., Cai R., Rui Q., Xi Z. (2017). miR-205 as a biological marker in non-small cell lung cancer. Biomed. Pharmacother..

[141] Kim J.H., Hwang C., Kim D.Y. (2025). Structurally Engineered All-Polymeric Microneedle Sensor for Highly Sensitive and Real-Time Monitoring of Glucose in Interstitial Fluids. ACS Sens..

[142] Zhang W., Xu J., Li X., Jang Y., Liu Q., Ma W., Luo G., Ma M., Wang Q., He Y. (2026). Augmenting ultrasound for continuous glucose monitoring via a wearable acoustically readable microneedle patch. Sci. Adv..

[143] Chien M.-N., Chen Y.-J., Bai C.-H., Huang J.-T. (2022). Continuous Glucose Monitoring System Based on Percutaneous Microneedle Array. Micromachines.

[144] Yang S., Qiao X., Ma J., Yang Z., Luo X., Du Z. (2025). Recent Advances in Flexible Sensors for Neural Interfaces: Multimodal Sensing, Signal Integration, and Closed-Loop Feedback. Biosensors.

[145] Xu M., Zhao Y., Xu G., Zhang Y., Sun S., Sun Y., Wang J., Pei R. (2022). Recent Development of Neural Microelectrodes with Dual-Mode Detection. Biosensors.

[146] Wang M., Zhou C., Ma J., Yin H., Wang Y., Shen H., Ge C., Zhou Y., Liu M., Liu Y. (2026). A neuron-scale all-hydrogel electrode array for chronic brain activity recording. Matter.

[147] Tong M., Pillai R.G., Kobryn A., Yan Z., Chan N.W.C., Jemere A.B. (2025). A Polydopamine-Based Molecularly Imprinted Electrochemical Sensor for Fentanyl Determination. ACS Omega.

[148] Li L., Zhou Y., Sun C., Zhou Z., Zhang J., Xu Y., Xiao X., Deng H., Zhong Y., Li G. (2024). Fully integrated wearable microneedle biosensing platform for wide-range and real-time continuous glucose monitoring. Acta Biomater..

[149] Ameri M., Shabaninejad Z., Movahedpour A., Sahebkar A., Mohammadi S., Hosseindoost S., Ebrahimi M.S., Savardashtaki A., Karimipour M., Mirzaei H. (2020). Biosensors for detection of Tau protein as an Alzheimer’s disease marker. Int. J. Biol. Macromol..

[150] Liu H., Chang S., Chen S., Du Y., Wang H., Wang C., Xiang Y., Wang Q., Li Z., Wang S. (2022). Highly sensitive and rapid detection of SARS-CoV-2 via a portable CRISPR-Cas13a-based lateral flow assay. J. Med. Virol..

[151] Zhang Y., Chai Y., Hu Z., Xu Z., Li M., Chen X., Yang C., Liu J. (2022). Recent Progress on Rapid Lateral Flow Assay-Based Early Diagnosis of COVID-19. Front. Bioeng. Biotechnol..

[152] Ho K.H.W., Lai H., Zhang R., Chen H., Yin W., Yan X., Xiao S., Lam C.Y.K., Gu Y., Yan J. (2024). SERS-Based Droplet Microfluidic Platform for Sensitive and High-Throughput Detection of Cancer Exosomes. ACS Sens..

[153] Li H., Fu X., You Q., Shi D., Su L., Song M., Peng R., Fu T., Wang P., Tan W. (2025). Multiple aptamer recognition-based quantum dot lateral flow platform: Ultrasensitive point-of-care testing of respiratory infectious diseases. J. Mater. Chem. B.

[154] He J., Zhu S., Zhou J., Jiang W., Yin L., Su L., Zhang X., Chen Q., Li X. (2023). Rapid detection of SARS-CoV-2: The gradual boom of lateral flow immunoassay. Front. Bioeng. Biotechnol..

[155] Chen B., Qiu X., Li Y. (2025). Exosomes in ovarian cancer: Impact on drug resistance and advances in SERS detection techniques. J. Pharm. Anal..

[156] Krage C., Adigüzel S., Thongrom B., Dimde M., Block S., Saeed M., Schulze M., Junge F., Klimek A., Achazi K. (2025). Three-Dimensional Polyglycerol-PEG-Based Hydrogels as a Universal High-Sensitivity Platform for SPR Analysis. Anal. Chem..

[157] Park S., Gerber A., Santa C., Aktug G., Hengerer B., Clark H.A., Jonas U., Dostalek J., Sergelen K. (2025). Molecularly Responsive Aptamer-Functionalized Hydrogel for Continuous Plasmonic Biomonitoring. J. Am. Chem. Soc..

[158] Wei X., Yin M., Zhang L., Lin H., Wang J., Xie W., Xu D. (2022). Surface Plasmon Resonance (SPR) biosensor for detection of mycotoxins: A review. J. Immunol. Methods.

[159] Wan H., Cui H., Liu H., Zhang G., He L., Liu H., Mahmud S. (2025). Surface plasmon resonance biosensor chips: Fabrications and pharmaceutical applications. J. Pharm. Biomed. Anal..

[160] Das S., Devireddy R., Gartia M.R. (2023). Surface Plasmon Resonance (SPR) Sensor for Cancer Biomarker Detection. Biosensors.

[161] Luo S., Wang R., Wang L., Qu H., Zheng L. (2022). Breath alcohol sensor based on hydrogel-gated graphene field-effect transistor. Biosens. Bioelectron..

[162] Yan Y., Yu L., Zhang X., Han Q., Yang Z., Lu X., Wang J., Xu H., Chen Q., Zhao H. (2025). Instantaneous self-recovery and ultra-low detection limit hydrogel electronic sensor for temporomandibular disorders intelligent diagnosis. Nat. Commun..

[163] Gao K., Xu K. (2025). Advancements and Prospects of pH-Responsive Hydrogels in Biomedicine. Gels.

[164] Arjama M., Mehnath S., Jeyaraj M. (2022). Self-assembled hydrogel nanocube for stimuli responsive drug delivery and tumor ablation by phototherapy against breast cancer. Int. J. Biol. Macromol..

[165] Tang H., Yang Y., Liu Z., Li W., Zhang Y., Huang Y., Kang T., Yu Y., Li N., Tian Y. (2024). Injectable ultrasonic sensor for wireless monitoring of intracranial signals. Nature.

[166] Saha S., Ali A., Agrawal T., Kalathil D.K., Gupta S.K., Rakshit T., Pal S. (2026). An in situ-forming oxidized alginate-boronate hydrogel for glucose-responsive insulin release in vivo. Mater. Adv..

[167] Shen H., Bao X., Qi B., Liang X., Chang Y., Hu X., Gao W., Sun D. (2025). Reactive oxygen species-responsive hydrogel loaded with melatonin as a minimally invasive treatment for myocardial I/R injury. iScience.

[168] Gu J., Zhao G., Yu J., Xu P., Yan J., Jin Z., Chen S., Wang Y., Zhang L.W., Wang Y. (2022). Injectable pH-responsive hydrogel for combinatorial chemoimmunotherapy tailored to the tumor microenvironment. J. Nanobiotechnology.

[169] Chen W., Li Z., Wang Z., Gao H., Ding J., He Z. (2020). Intraarticular Injection of Infliximab-Loaded Thermosensitive Hydrogel Alleviates Pain and Protects Cartilage in Rheumatoid Arthritis. J. Pain Res..

[170] Lian H., Liu L., Ke L. (2025). Current Research Status of Biomedical Hydrogel and Challenges and Opportunities in Clinical Translation. Chin. J. Med. Instrum..

[171] Quazi M.Z., Hwang J., Song Y., Park N. (2023). Hydrogel-Based Biosensors for Effective Therapeutics. Gels.

[172] Wang Y., Gao Y., Tang L., Guo Y., Sha B., Jiang Y. (2026). Hydrogel-based wearable and implantable biosensors in health monitoring. Biomater. Sci..

[173] Chimene D., Queener K.M.K., Ko B.S., McShane M., Daniele M. (2024). Insertable Biosensors: Combining Implanted Sensing Materials with Wearable Monitors. Annu. Rev. Biomed. Eng..

[174] Lee M.A., Jin X., Muthupalani S., Bakh N.A., Gong X., Strano M.S. (2023). In-Vivo fluorescent nanosensor implants based on hydrogel-encapsulation: Investigating the inflammation and the foreign-body response. J. Nanobiotechnology.

[175] Amani H., Alipour M., Shahriari E., Taboas J.M. (2024). Immunomodulatory Biomaterials: Tailoring Surface Properties to Mitigate Foreign Body Reaction and Enhance Tissue Regeneration. Adv. Healthc. Mater..

[176] Zhou X., Cao W., Chen Y., Zhu Z., Lai Y., Liu Z., Jia F., Lu Z., Han H., Yao K. (2024). An elastomer with in situ generated pure zwitterionic surfaces for fibrosis-resistant implants. Acta Biomater..

[177] Wang Z., Sun Y., Li C. (2024). Advances in 3D printing technology for preparing bone tissue engineering scaffolds from biodegradable materials. Front. Bioeng. Biotechnol..

[178] Lv Z., Peng B., Ye Y., Xu H., Cai X., Liu J., Dai J., Bian Y., Wen P., Weng X. (2025). Bolstered bone regeneration by multiscale customized magnesium scaffolds with hierarchical structures and tempered degradation. Bioact. Mater..

[179] Xu X., Zhang J., Filion T.M., Akalin A., Song J. (2021). Modulating Mechanical and Shape-Memory Properties while Mitigating Degradation-Induced Inflammation of Polylactides by Pendant Aspirin Incorporation. ACS Appl. Mater. Interfaces.

[180] Manavitehrani I., Fathi A., Badr H., Daly S., Negahi Shirazi A., Dehghani F. (2016). Biomedical Applications of Biodegradable Polyesters. Polymers.

[181] Sun Han Chang R.A., Shanley J.F., Kersh M.E., Harley B.A.C. (2020). Tough and tunable scaffold-hydrogel composite biomaterial for soft-to-hard musculoskeletal tissue interfaces. Sci. Adv..

[182] Zhuang P., Chen L., Zhang Y., Yang W., Chen Y., Wu L., Xiang L., Wang Z., Rosenholm J.M., Zuo G. (2025). Solid-Liquid Interface Lubricating Hydrogels for Tendon-Bone Healing. Research.

[183] Barhoum A., Sadak O., Ramirez I.A., Iverson N. (2023). Stimuli-bioresponsive hydrogels as new generation materials for implantable, wearable, and disposable biosensors for medical diagnostics: Principles, opportunities, and challenges. Adv. Colloid Interface Sci..

[184] Du J., Ma Q., Wang B., Sun L., Liu L. (2023). Hydrogel fibers for wearable sensors and soft actuators. iScience.

[185] Yao M., Hsieh J.-C., Wang H. (2026). Hydrogel-integrated multimodal physiological and modulation systems. Mater. Horiz..

[186] Zhao Y., Cui J., Qiu X., Yan Y., Zhang Z., Fang K., Yang Y., Zhang X., Huang J. (2022). Manufacturing and post-engineering strategies of hydrogel actuators and sensors: From materials to interfaces. Adv. Colloid Interface Sci..

[187] Park J.Y., Barrera N., Bai T., Meng E., Fang H., Lee H. (2025). Lessons Learned and Challenges Ahead in the Translation of Implantable Microscale Sensors and Actuators. Annu. Rev. Biomed. Eng..

[188] Jain I., Sellers G.S., Jain A., Agarwal A., Davé R.S. (2025). Chapter Three-The ethical and regulatory landscape of wearable, ingestible, and implantable technologies in the United States. Biosensing the Future: Wearable, Ingestible and Implantable Technologies for Health and Wellness Monitoring Part B.

